# Supplementary figures and images for: Ortholog of autism candidate gene RBM27 regulates mitoribosomal assembly factor MALS-1 to protect against mitochondrial dysfunction and axon degeneration during neurodevelopment
Source: PLoS Biol. 2024 Oct 31;22(10):e3002876. doi: 10.1371/journal.pbio.3002876 (PMC11556708; doi:10.1371/journal.pbio.3002876)

RBM-26 3XFLAG tagged variants

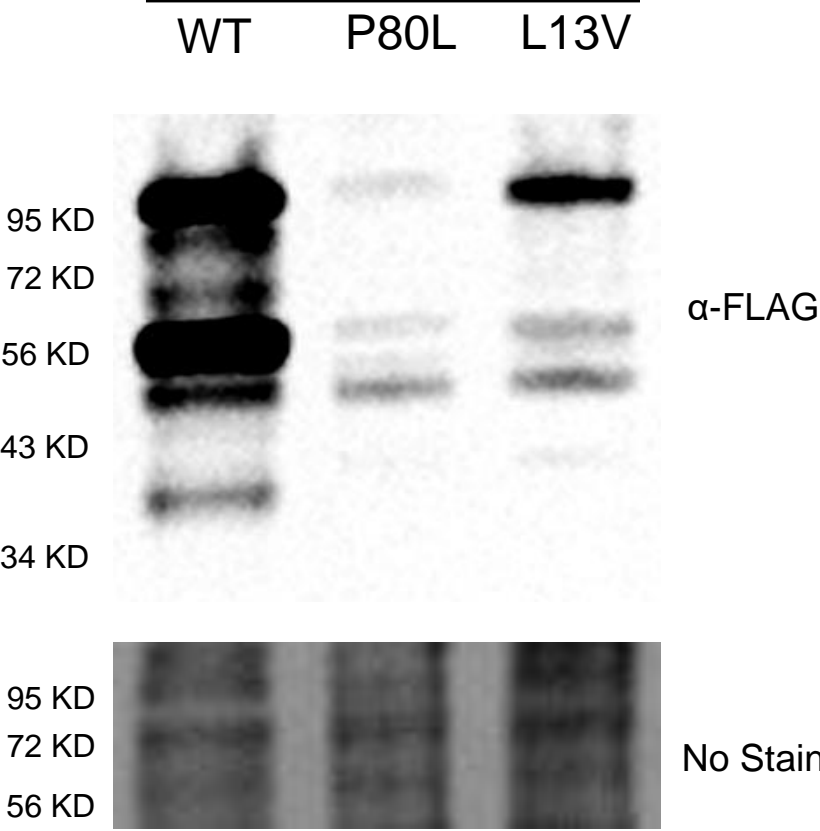

Supplement: S1 Fig — Representative western blot of 3 biological replicates showing expression of RBM-26::3XFLAG, RBM-26 P80L::3XFLAG, and RBM-26 L13V::3XFLAG proteins. A total of 20 μg of total protein lysate was loaded per well and specific proteins were detected with an anti-FLAG antibody and enhanced chemiluminescence. Protein loading was quantified by No-Stain Protein Labeling Reagent. (PDF) [file pbio.3002876.s001.pdf]

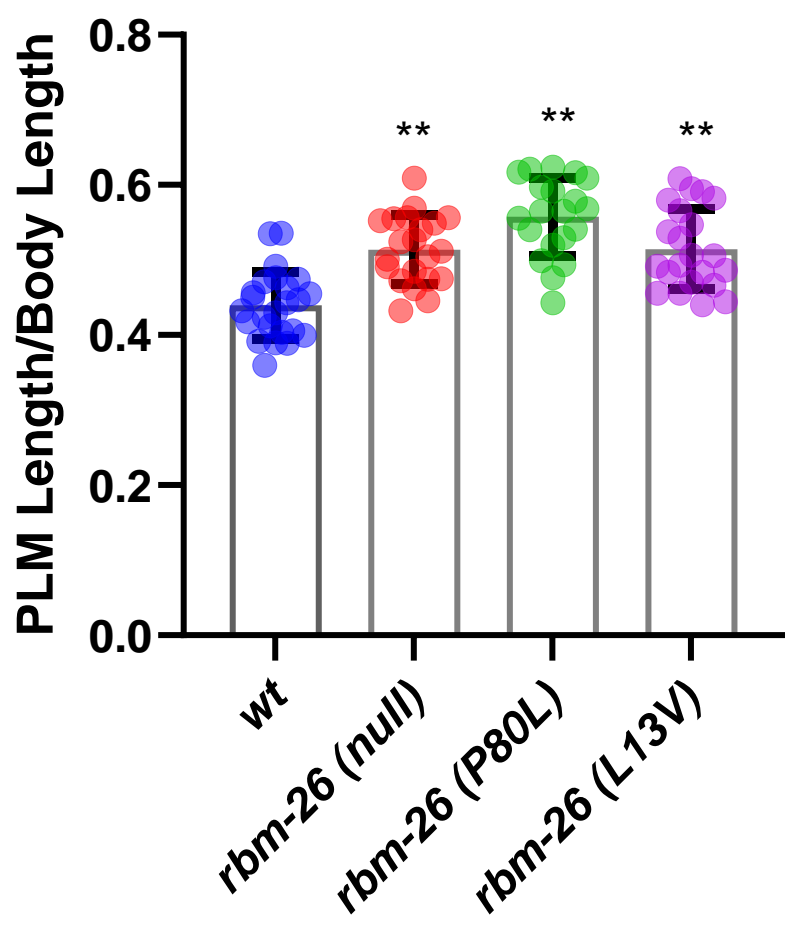

Supplement: S2 Fig — PLM length as a ratio of body length at L3 in wild type (wt), rbm-26 (null), rbm-26 (P80L), and rbm-26 (L13V) mutants. Error bars = SD. Statistical significance was analyzed by one-way ANOVA with a Tukey post hoc test, ** p < 0.01. n for wt = 24, n for rbm-26 null = 21, n for rbm-26 (P80L) = 20, and n for rbm-26 (L13V) = 22. Alleles: rbm-26(null) is rbm-26(gk910); rbm-26(P80L) is rbm-26(cue23); rbm-26(L13V) is rbm-26(cue34). Underlying data can be found in S2 Data. (PDF) [file pbio.3002876.s002.pdf]

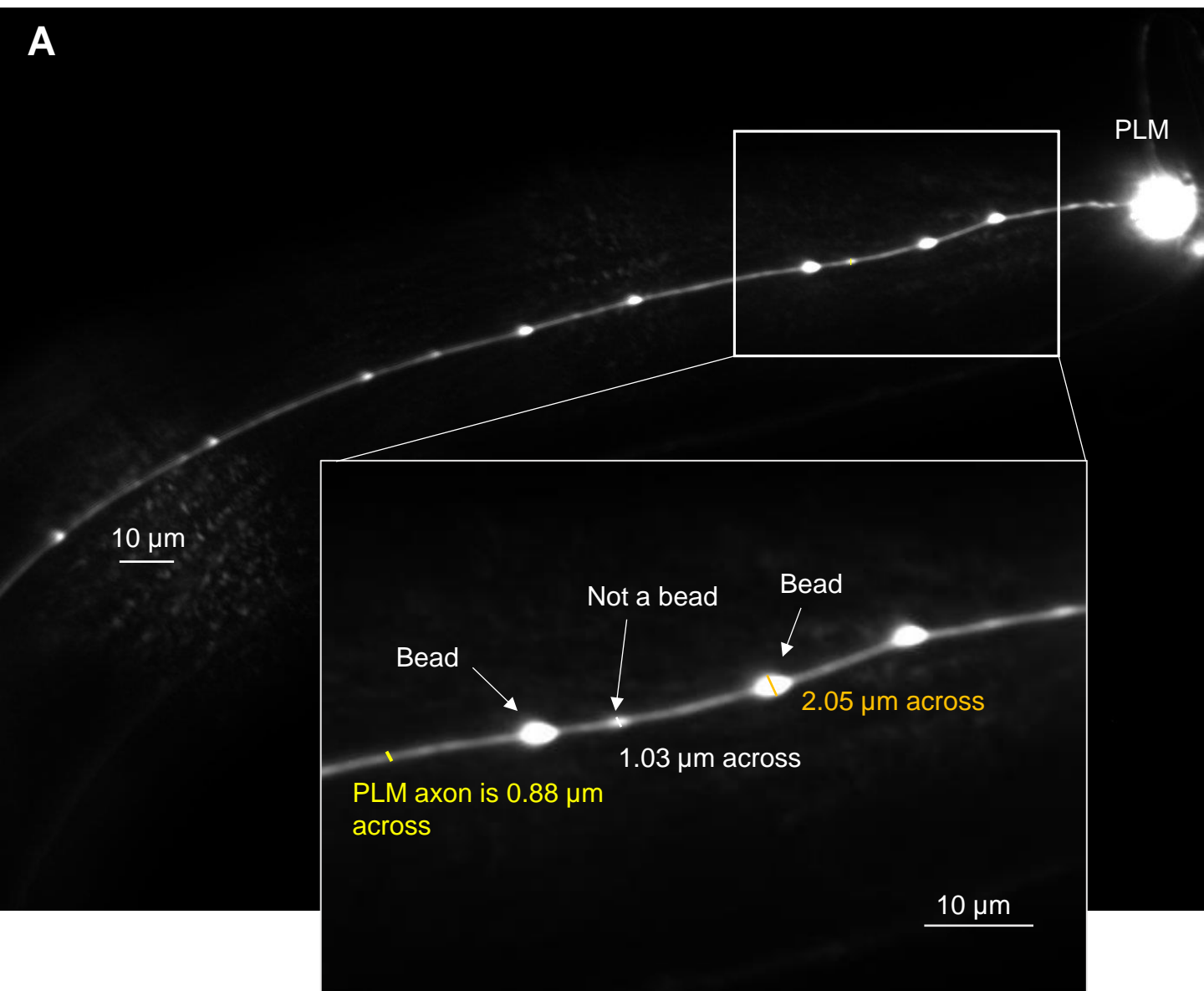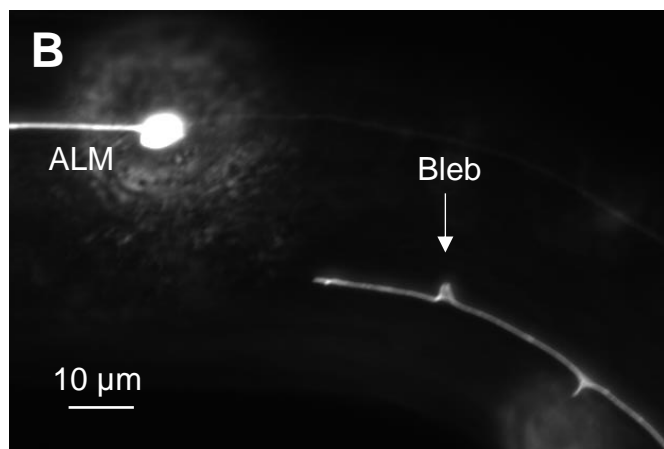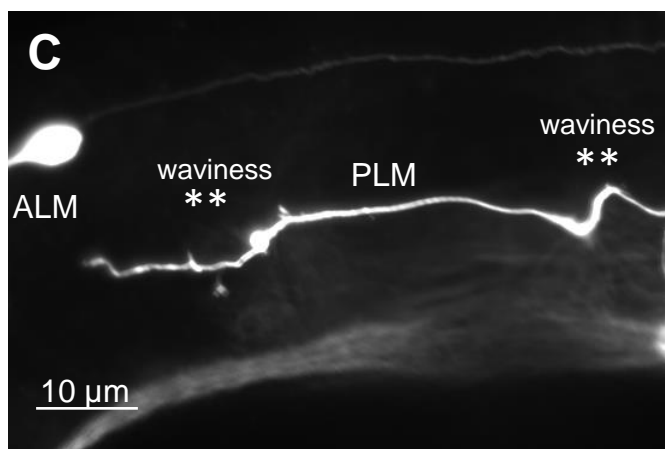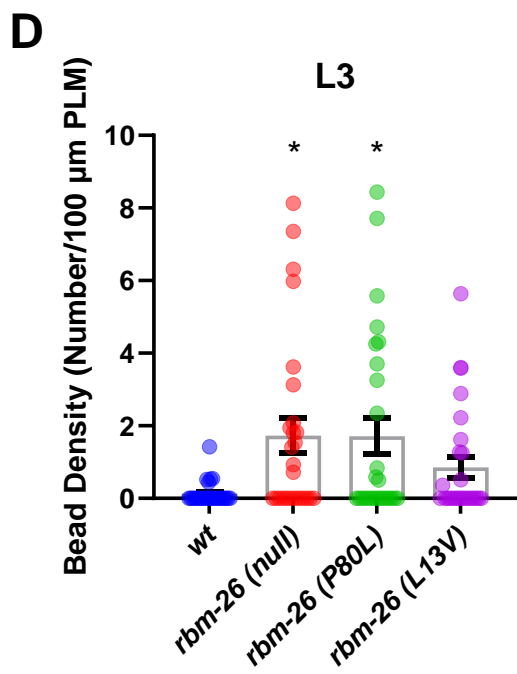

Supplement: S3 Fig — (A) Example of beading phenotype. Only those focal enlargements along PLM axon that were about twice the diameter of the axon were considered to be beads. (B) Example of blebbing phenotype. (C) Example of waviness in PLM axon. (D) Quantification of the number of beads per 100 μm of PLM axon (bead density) observed in wild type (wt), rbm-26 (null), rbm-26 (P80L), and rbm-26 (L13V) at L3. Error bars are standard error of mean. Statistical significance was analyzed by one-way ANOVA with a Tukey post hoc test, * p < 0.05 (n = 28). Underlying data can be found in S2 Data. (PDF) [file pbio.3002876.s003.pdf]

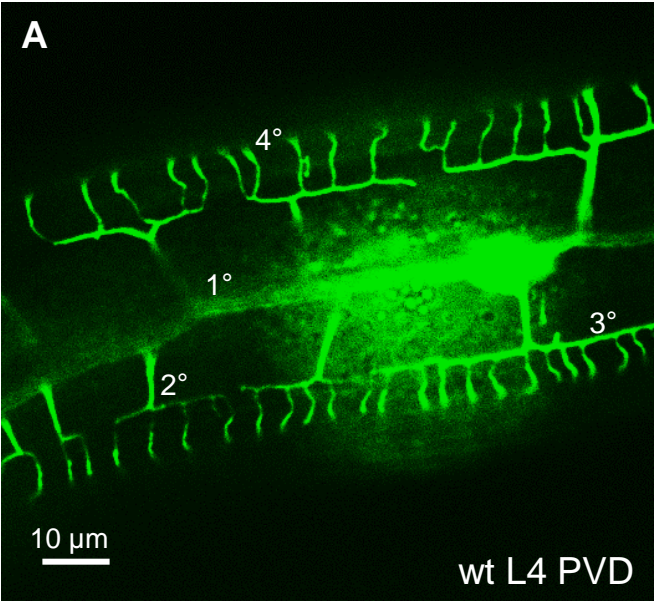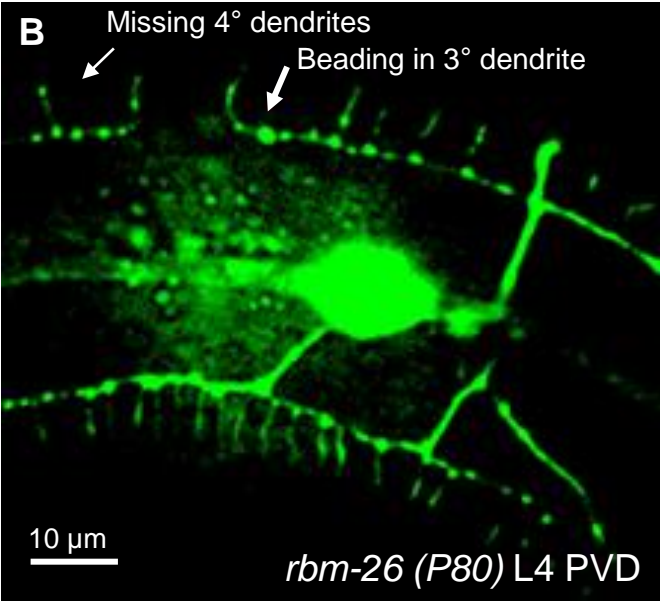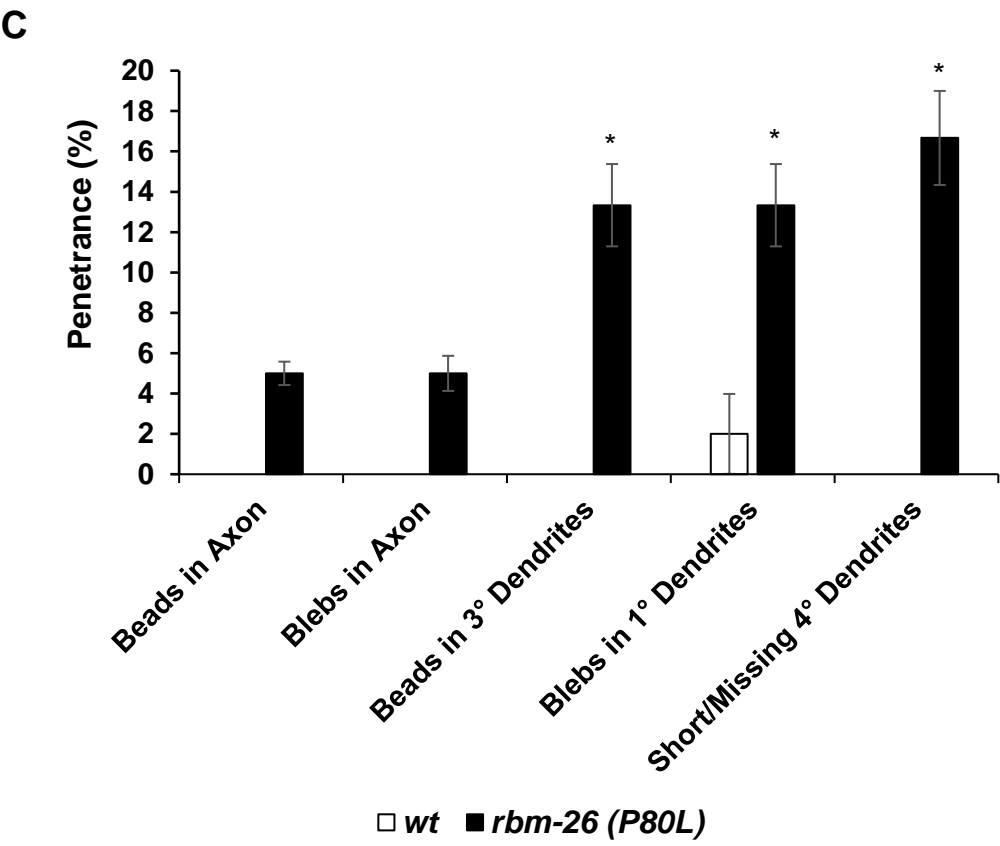

Supplement: S4 Fig — (A) Wild-type L4 PVD–primary (1°), secondary (2°), tertiary (3°), and quaternary (4°) dendrites can be seen. (B) Beading can be seen in tertiary dendrites and some quaternary dendrites are missing in rbm-26 (P80L) L4 PVD neuron. (C) Quantification of phenotypes observed in rbm-26 (P80L) mutants, and 50 wild-type L4 PVD neurons were observed and 60 rbm-26 (P80L) L4 PVD neurons were observed. PVD neurons were visualized by the wdIs52 [F49H12.4::GFP + unc-119(+)] transgene, which expresses GFP in PVD. Asterisks indicate statistically significant difference relative to wild type, Z-test for proportions (* p < 0.05), and error bars represent the standard error of the proportion. Underlying data can be found in S2 Data. (PDF) [file pbio.3002876.s004.pdf]

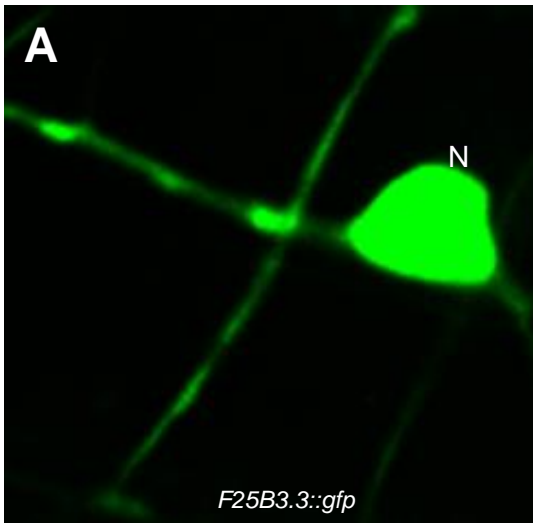

Neuron

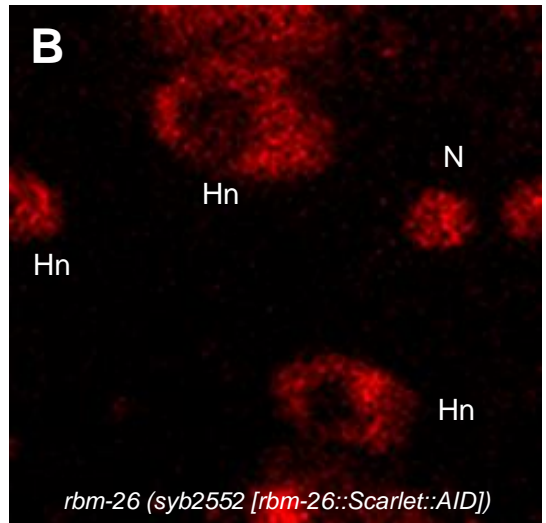

RBM-26::Scarlet

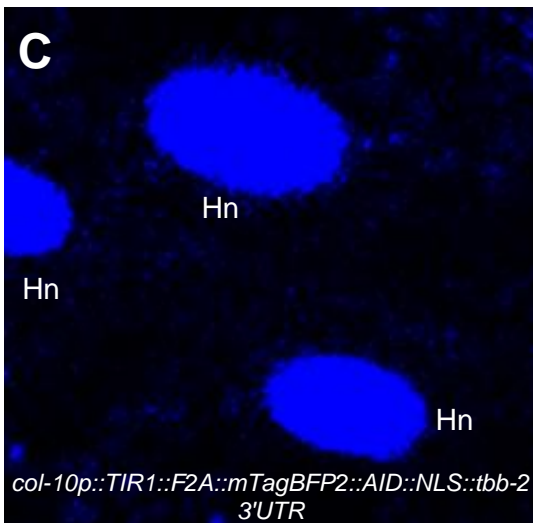

Hypodermis

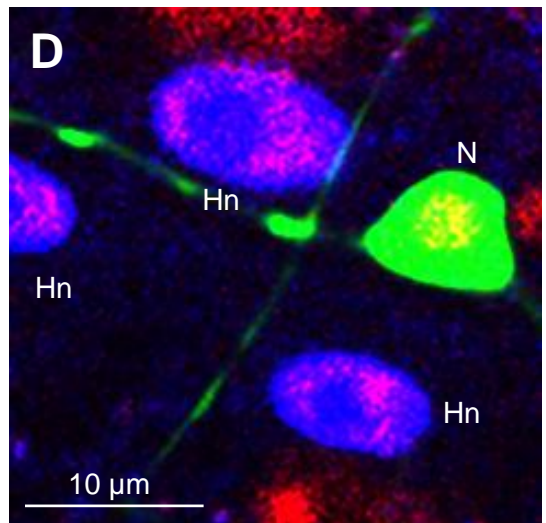

Merged

Supplement: S5 Fig — RBM-26 is expressed in the nuclei of neurons and hypodermal cells. Neurons are identified by the pan-neuronal expression of GFP by the evIs111 transgene (panels A and D). Hypodermal nuclei are identified by tissue-specific expression of blue fluorescence protein tagged with a nuclear localization signal and an auxin-induced degron (col-10p::TIR1::F2A::mTagBFP2::AID::NLS::tbb-2 3’ UTR) (panels C and D). Panels B and D show endogenously tagged RBM-26 (rbm-26::Scarlet::AID). Panels A–C are merged in D. “N” in panels A, B, and D indicate neuron and “Hn” in panels B, C, and D indicate hypodermal nuclei. (PDF) [file pbio.3002876.s005.pdf]

RBM-26::Scarlet (*wt*) L3

Head

Tail

10μm

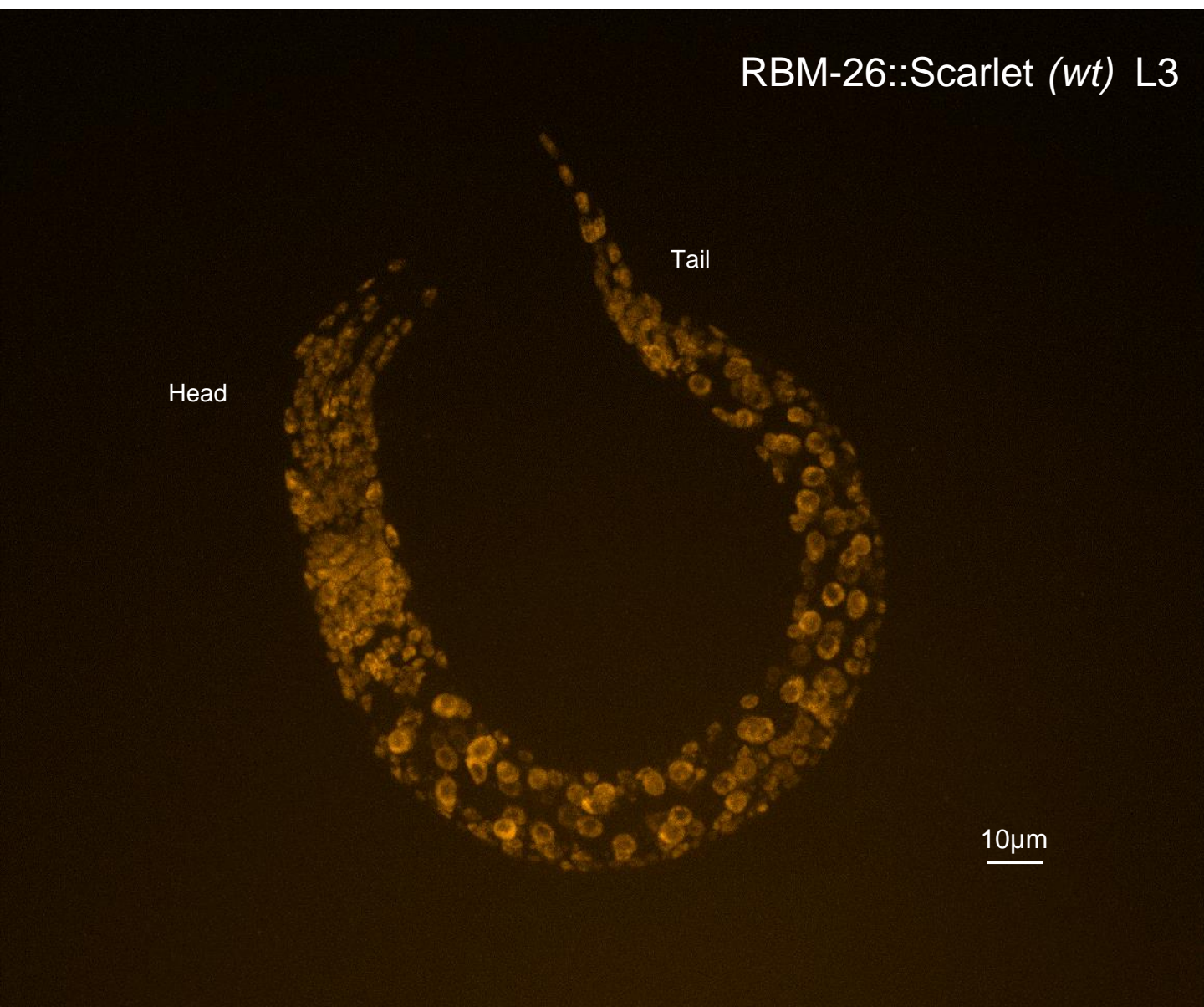

Supplement: S6 Fig — Representative Z-stack projection of endogenous RBM-26 protein tagged with wormScarlet (RBM-26::SCARLET::AID) at larval stage L3. (PDF) [file pbio.3002876.s006.pdf]

**A**

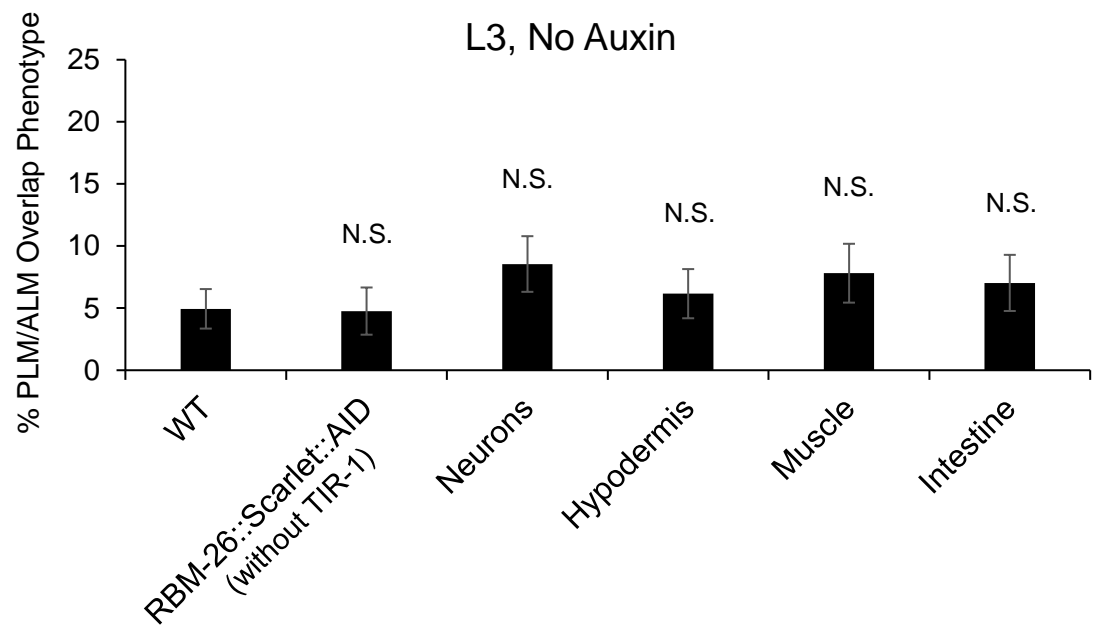

**B**

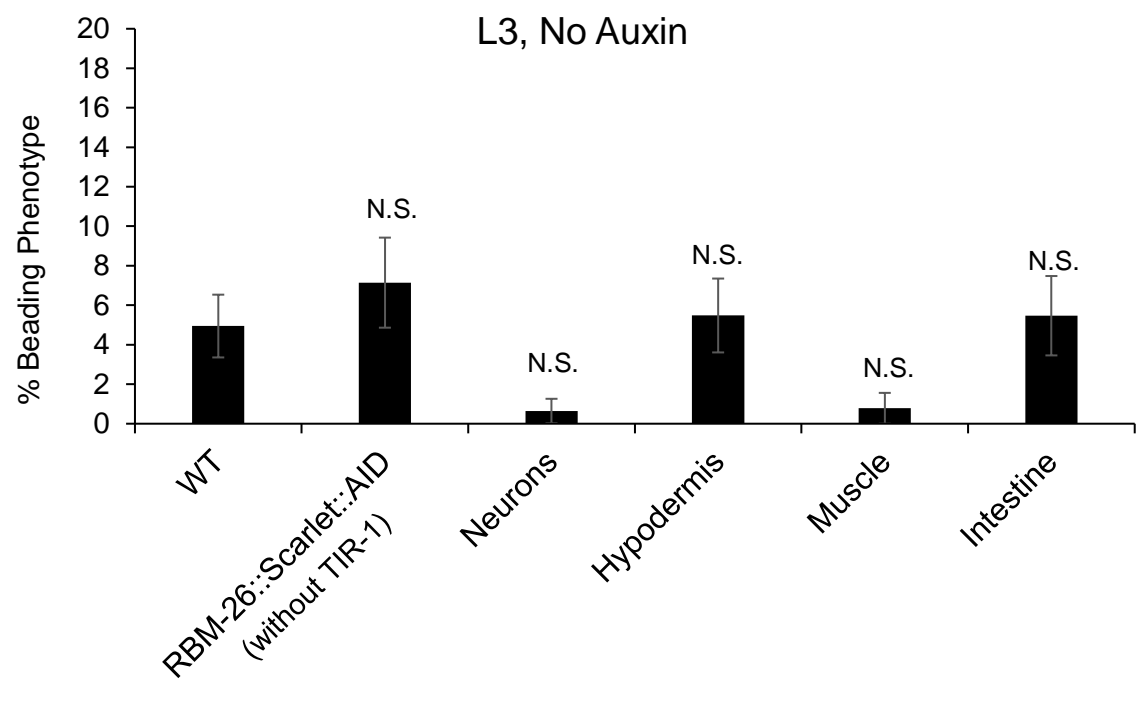

Supplement: S7 Fig — Worms expressing auxin inducible degron (AID) in frame with RBM-26 and tissue-specific TIR-1 as indicated on the X-axis were placed in Auxin free worm plates at L4 and their progeny were observed at L3; “n” is between 125 and 150. N.S. = Not Significant (Z-test for proportions). Error bars represent the standard error of the proportion. Underlying data can be found in S2 Data. (PDF) [file pbio.3002876.s007.pdf]

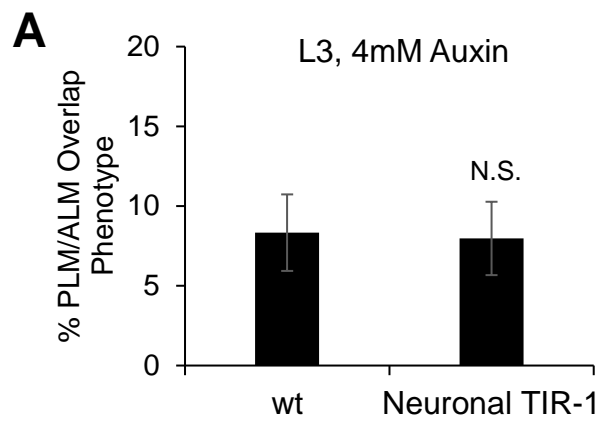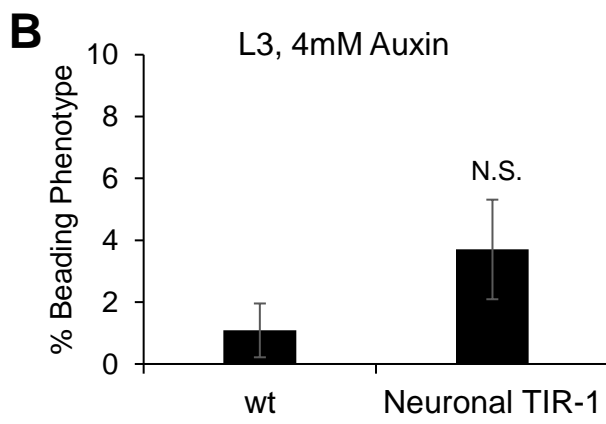

Supplement: S8 Fig — L4 Wild type or Neuronal TIR-1 (without AID tagged RBM-26) were placed on 4 mM synthetic Auxin-coated plates, allowed to lay eggs and their L3 progenies were analyzed for (A) PLM termination defect and (B) beading phenotype; “n” is 132 for wt and 138 for Neuronal TIR-1. N.S. = Not Significant (Z-test for proportions). Error bars represent the standard error of the proportion. Underlying data can be found in S2 Data. (PDF) [file pbio.3002876.s008.pdf]

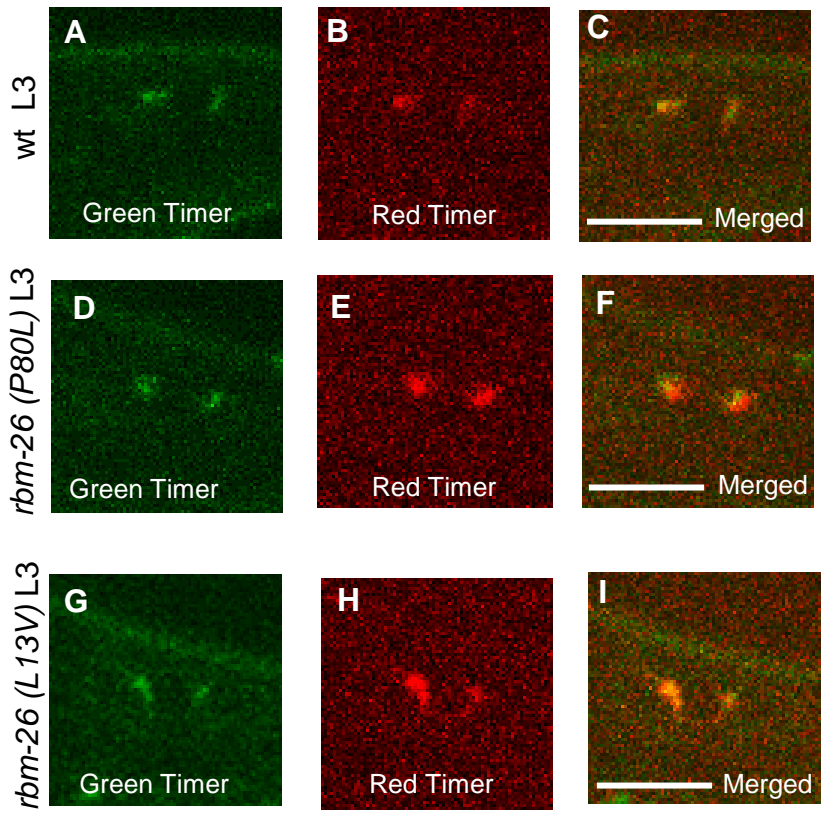

Supplement: S9 Fig — mitoTimer is identified by the transgene cueSi35(Pmec-7::mitoTimer::tbb-2 3′ UTR). rbm-26(P80L) is rbm-26(cue23); rbm-26(L13V) is rbm-26(cue34). Scale bars are 5 μm. (PDF) [file pbio.3002876.s009.pdf]

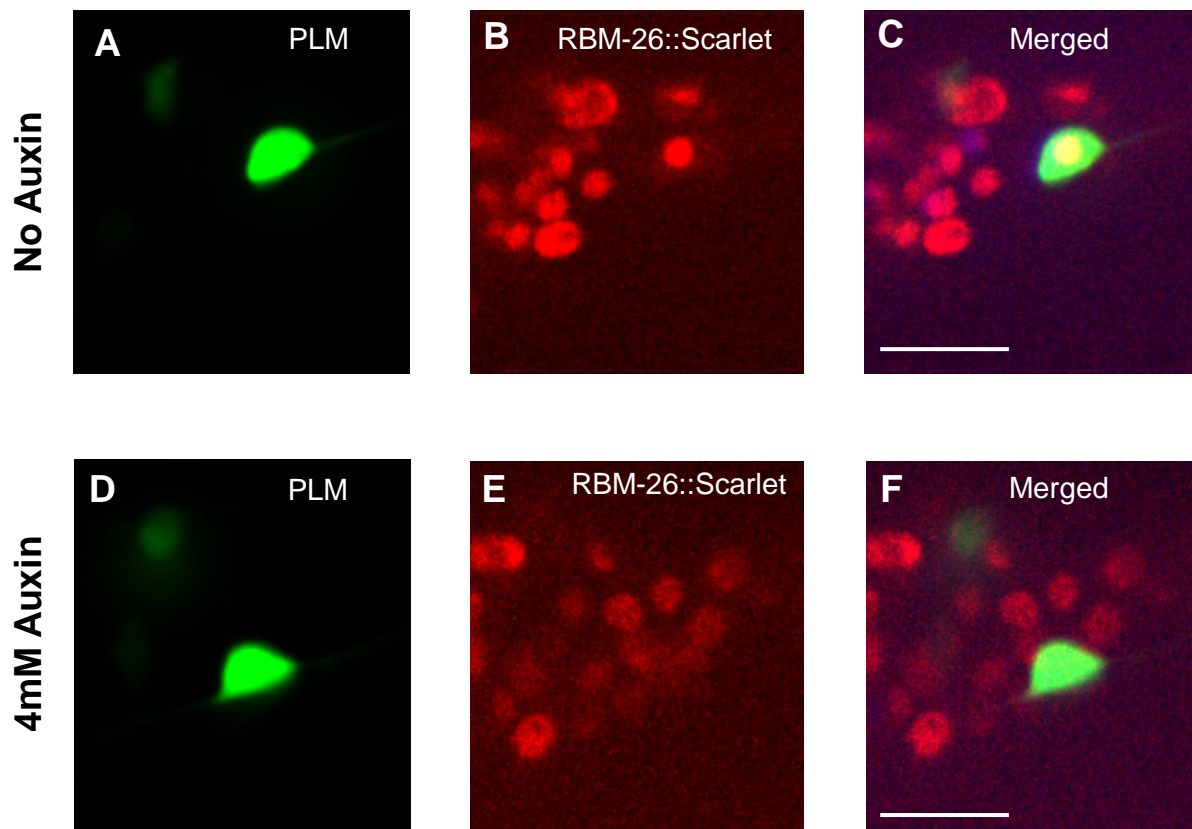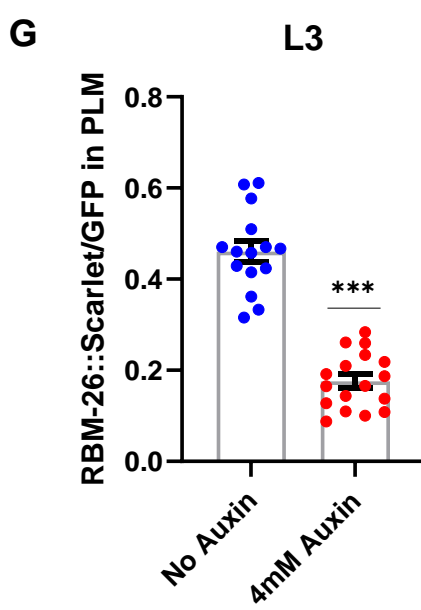

Supplement: S10 Fig — L4 rbm-26 (syb2552); resi7 worms that express RBM-26::Scarlet::AID and F-box Transport Inhibitor Response 1 (TIR-1) in neurons were placed in normal NGM plates (A–C) or on plates coated with 4 mM synthetic auxin (D, E), allowed to lay eggs and L3 progenies were analyzed through confocal microscopy. PLM neuron was visualized with the help of the muIs32 (Pmec-7::gfp) transgene. Scale bars are 10 μm. Auxin treatment causes depletion of RBM-26 protein, which was quantified in G as a ratio of RBM-26::Scarlet intensity relative to the GFP expression in PLM. Underlying data can be found in S2 Data. (PDF) [file pbio.3002876.s010.pdf]

RNA standards used for quantifying *mals-1* mRNA levels

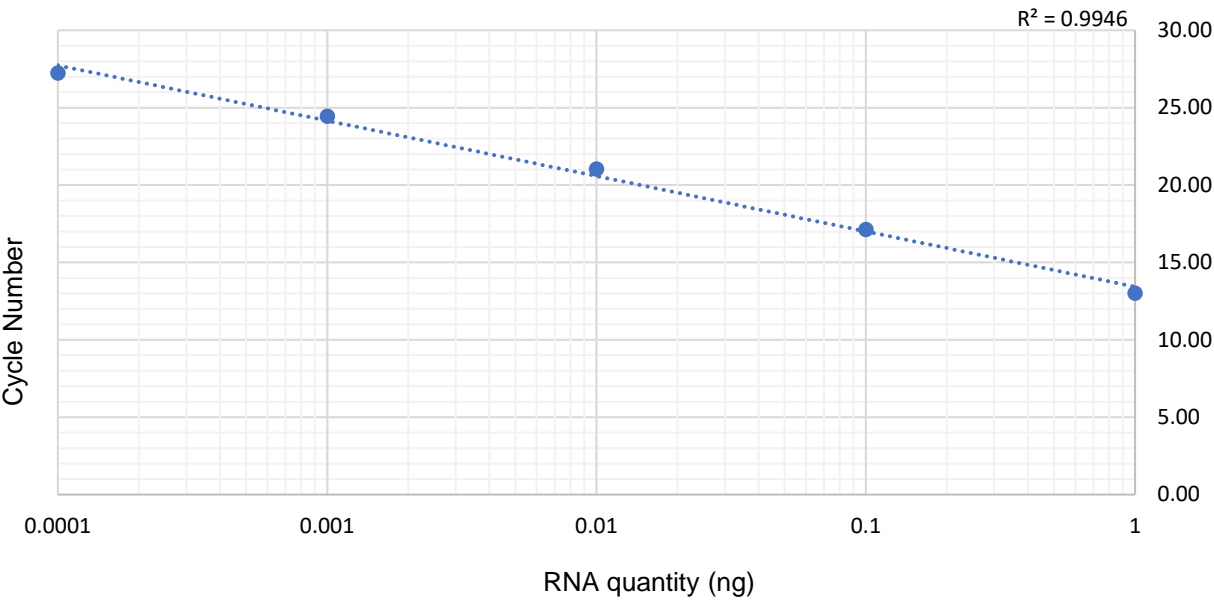

Supplement: S11 Fig — Synthetic RNA corresponding to 68 nucleotides in the last exon and the 3′ UTR of mals-1 mRNA was purchased from Azenta Inc. The RNA was diluted 1 ng/μl, 0.1 ng/μl, 0.01 ng/μl, 0.001 ng/μl, and 0.0001 ng/μl in nuclease free water. The dilutions were quantified in Qubit (Thermo Fisher Scientific) using RNA Qubit Broad Range kit (Thermo Fischer Scientific); 1, 0.1, 0.01, 0.001, and 0.0001 ng of RNA standard was used in RT-qPCR reaction. Cycle to threshold (Ct) values obtained from RT-qPCR reaction were plotted against the known quantity of RNA used. (PDF) [file pbio.3002876.s011.pdf]
