# Supplementary material for: Ortholog of autism candidate gene RBM27 regulates mitoribosomal assembly factor MALS-1 to protect against mitochondrial dysfunction and axon degeneration during neurodevelopment
Source: PLoS Biol. 2024 Oct 31;22(10):e3002876. doi: 10.1371/journal.pbio.3002876 (PMC11556708; doi:10.1371/journal.pbio.3002876)
Supplement: S1 Table — Maternally rescued rbm-26 (null) worms do not survive past L3. Asterisks indicate statistically significant difference relative to wild type, Z-test for proportions (*** p < 0.0001, ** p < 0.01, and * p < 0.05) while “ns” indicates no significant difference. (PDF) [file pbio.3002876.s012.pdf]

Genotype	Age	% Blebbing	% Beading	% Waviness	% Break	N
Wild type	L1	0.925926	1.851852	0	0	108
Wild type	L2	0	2.941176	0.980392	0	102
Wild type	L3	0	4.545455	1.515152	0	198
Wild type	L4	0	6.097561	1.829268	0	164
Wild type	1-DAY Adult	0	14.03509	4.385965	0	114
Wild type	2-DAY Adult	1.886792	17.92453	0	0.943396	106
rbm-26 (null)	L1	5^{ns}	0	0	0	80
rbm-26 (null)	L2	2.631579*	11.40351*	1.754386^{ns}	0	114
rbm-26 (null)	L3	0	32.36715***	13.52657***	1.449275^{ns}	207
rbm-26 (null)	L4	NA	NA	NA	NA	NA
rbm-26 (null)	1-DAY Adult	NA	NA	NA	NA	NA
rbm-26 (null)	2-DAY Adult	NA	NA	NA	NA	NA
rbm-26 (P80L)	L1	1.960784^{ns}	8.823529^{ns}	3.921569*	0	102
rbm-26 (P80L)	L2	1.6^{ns}	15.2**	1.6^{ns}	0	125
rbm-26 (P80L)	L3	0	24.37811***	7.462687***	1.492537^{ns}	201
rbm-26 (P80L)	L4	2.112676^{ns}	26.40845***	2.112676^{ns}	2.112676^{ns}	284
rbm-26 (P80L)	1-DAY Adult	2.542373^{ns}	33.89831***	4.237288^{ns}	1.694915^{ns}	118
rbm-26 (P80L)	2-DAY Adult	3.448276^{ns}	62.6506***	4.237288*	6.024096*	118
rbm-26 (L13V)	L1	0.980392^{ns}	10.78431^{ns}	2.941176^{ns}	0	102
rbm-26 (L13V)	L2	2^{ns}	8.783784^{ns}	0.666667^{ns}	0	150
rbm-26 (L13V)	L3	1.470588^{ns}	13.23529**	3.333333^{ns}	0	204
rbm-26 (L13V)	L4	0.78125^{ns}	13.88889*	2.5^{ns}	0	128
rbm-26 (L13V)	1-DAY Adult	0.847458^{ns}	24.57627***	3.389831^{ns}	0.847458^{ns}	118
rbm-26 (L13V)	2-DAY Adult	3.508772^{ns}	36.84211***	3.508772^{ns}	2.631579^{ns}	132
