# Supplementary material for: Ortholog of autism candidate gene RBM27 regulates mitoribosomal assembly factor MALS-1 to protect against mitochondrial dysfunction and axon degeneration during neurodevelopment
Source: PLoS Biol. 2024 Oct 31;22(10):e3002876. doi: 10.1371/journal.pbio.3002876 (PMC11556708; doi:10.1371/journal.pbio.3002876)
Supplement: S2 Table — (PDF) [file pbio.3002876.s013.pdf]

Gene.name	Human Ortholog (HGNC Symbol)[1]	log2FoldChange	padj
F59C6.15	NA[2]	3.529339	1.70E-09
pud-2.1	NA	3.103615	5.63E-06
pud-2.2	NA	3.04405	7.45E-06
pud-1.1	NA	2.772255	2.98E-05
pud-1.2	NA	2.749208	3.21E-05
C23H5.8	NA	2.578636	0.00021
C37A2.9	NA	5.056751	0.000568
ilys-5	NA	2.848721	0.000745
K12H4.2 (mals-1)	MALSU1	1.983865	0.000826
rrn-2.1	NA	2.319809	0.002424
clec-85	NA	1.860425	0.00272
K07C5.11	NA	2.422354	0.002764
21ur-8178	NA	4.814433	0.003612
spp-4	NA	2.183194	0.005335
Y6B3B.13	NA	3.736574	0.00653
21ur-3462	NA	4.815693	0.009799
K02A11.4	NA	2.023582	0.011462
21ur-10303	NA	4.592398	0.01198
F43A11.8	NA	2.201064	0.01198
F33H12.7	NA	2.189781	0.014145
msp-81	NA	1.939555	0.01655
fip-7	NA	2.878975	0.017713
21ur-12103	NA	4.77819	0.019219
Y46G5A.40	NA	2.514422	0.021423
F55A8.4	NA	1.86247	0.022547
clc-1	NA	1.772934	0.023129
clec-209	NA	1.99952	0.026991
21ur-7931	NA	4.59147	0.027738
F59D8.3	NA	2.344256	0.027738
F22D6.16	NA	2.290677	0.027738
K07C5.18	NA	2.283691	0.027738
K06A4.7	NA	2.239518	0.027738
T19C4.17	NA	1.972172	0.027738
D1065.7	NA	2.118396	0.028351
C33H5.13	NA	1.929031	0.028351
dpy-4	MARCO	1.922891	0.028351
Y75B8A.28	NA	1.860966	0.028351
F28H7.3	NA	1.968765	0.029705
Y38H6C.23	NA	2.002049	0.029819
Y40B1A.5	NA	2.581652	0.030633
T27A10.8	NA	2.290521	0.031615
C08F11.116	NA	2.249682	0.032408
F16H6.10	NA	1.354888	0.032408
F56A4.2	NA	1.954246	0.035992
Y43F8C.22	NA	2.149066	0.03674
gst-22	GSTA1/GSTA2/GSTA3/GSTA4/GSTA5	1.583856	0.037515
W05H9.3	NA	1.974427	0.039296
F07H5.3	NA	1.942375	0.040116
F54B11.14	NA	1.465389	0.040116
C05E7.t2	NA	3.735348	0.041196
W02F12.11	NA	3.084148	0.041196
T19C9.8	NA	1.693645	0.041196
acdh-1	ACADS/ACADSB	2.136673	0.043721
lys-4	NA	2.048744	0.043721
21ur-7165	NA	4.475939	0.04407
F18C5.15	NA	2.463115	0.04407
H04D03.5	NA	2.223033	0.04407
JC8.15	NA	2.10709	0.04407
21ur-5454	NA	3.776464	0.045548
21ur-10438	NA	2.263752	0.045548
C18E9.14	NA	2.055965	0.046461
ZK546.18	NA	2.109542	0.049805
rme-2	LRP3/LRP12/LDLRAD3/LRP10	1.706674	0.049881

[1]Human orthologs were identified with Ortholist 2 (http://ortholist.shaye-lab.org/) [1].
[2]No human ortholog was found.

References

1. Kim W, Underwood RS, Greenwald I, Shaye DD. OrthoList 2: A New Comparative Genomic Analysis of Human and Caenorhabditis elegans Genes. Genetics. 2018;210(2):445-61. Epub 2018/08/19. doi: 10.1534/genetics.118.301307. PubMed PMID: 30120140; PubMed Central PMCID: PMCPMC6216590.
