# Supplementary material for: Ortholog of autism candidate gene RBM27 regulates mitoribosomal assembly factor MALS-1 to protect against mitochondrial dysfunction and axon degeneration during neurodevelopment
Source: PLoS Biol. 2024 Oct 31;22(10):e3002876. doi: 10.1371/journal.pbio.3002876 (PMC11556708; doi:10.1371/journal.pbio.3002876)
Supplement: S3 Table — (PDF) [file pbio.3002876.s014.pdf]

Genotype	Source
rbm-26 (cue23 [rbm-26 (P80L)]) III	this study
rbm-26 (cue34 [rbm-26 (L13V)]) III	this study
rbm-26 (cue25 [rbm-26::3X FLAG]) III	this study
rbm-26 (cue22 [rbm-26 (P80L)] cue25 [rbm-26::3X FLAG]) III	this study
rbm-26 (cue24 [rbm-26 (L13V)] cue25 [rbm-26::3X FLAG]) III	this study
mals-1 (cue37 [mals-1::3X FLAG]) III	this study
mals-1 (syb6330) III	SUNY biotech
rbm-26 (cue40 [rbm-26 (P80L)]) mals-1 (syb6330) III	this study
rbm-26 (cue42 [rbm-26 (P80L)]) mals-1 (cue37 [mals-1::3X FLAG]) III	this study
mals-1 (tm12122) III	Mitani lab, International *C. elegans* Gene Knockout Consortium
rbm-26 (cue 41 [rbm-26 (P80L)]) mals-1 (tm12122) III	This study
mrpl-58 (cue38 [mrpl-58::3XFLAG]) III	This study
rbm-26 (cue39 [rbm-26 (P80L)]) mrpl-58 (cue 38 [mrpl-58::3X FLAG]) III	This study
rbm-26 (cue 47 [rbm-26 (L13V)]) mals-1 (syb6330)] III	This study
rbm-26 (syb2552 [rbm-26::Scarlet::AID]) III	SUNY biotech
rbm-26 (cue 48 [rbm-26 (P80L)]) syb2552 [rbm-26::Scarlet::AID]) III	This study
cueSi35 (Pmec-7::mitoTimer::tbb-2 3'UTR) IV	This study
cueSi36 (Pmec-7::mals-1::scarlet::tbb-2 3'UTR) IV	This study
evIs111 [F25B3.3::GFP + dpy-20(+)]; rbm-26 (syb2552 [rbm-26::Scarlet::AID]) III; reSi1 [col-10p::TIR1::F2A::mTagBFP2::AID::NLS::tbb-2 3'UTR] I*	This study
muIs32 [mec-7p::GFP + lin-15(+)]	CGC
muIs32 [mec-7p::GFP + lin-15 (+)]; rbm-26 (cue23 [rbm-26 (P80L)]) III	this study
muIs32 [mec-7p::GFP + lin-15 (+)]; rbm-26 (gk910) III/hT2 [bli-4(e937) let-?(q782) qIs48] I;III	this study
muIs32 [mec-7p::GFP + lin-15 (+)]; rbm-26 (cue34 [rbm-26 (L13V)]) III	this study
muIs32 [mec-7p::GFP + lin-15(+)]; rbm-26 (syb2552 [rbm-26::Scarlet::AID]) III	this study
muIs32 [mec-7p::GFP + lin-15(+)]; rbm-26 (syb2552 [rbm-26::Scarlet::AID]) III; reSi1 [col-10p::TIR1::F2A::mTagBFP2::AID::NLS::tbb-2 3'UTR] I*	this study
muIs32 [mec-7p::GFP + lin-15(+)]; rbm-26 (syb2552 [rbm-26::Scarlet::AID]) III; reSi7 [rgef-1p::TIR1::F2A::mTagBFP2::AID::NLS::tbb-2 3'UTR] I*	this study
muIs32 [mec-7p::GFP + lin-15(+)]; rbm-26 (syb2552 [rbm-26::Scarlet::AID]) III; reSi3 [unc-54p::TIR1::F2A::mTagBFP2::AID::NLS::tbb-2 3'UTR] I*	this study
muIs32 [mec-7p::GFP + lin-15(+)]; rbm-26 (syb2552 [rbm-26::Scarlet::AID]) III; reSi12 [ges-1p::TIR1::F2A::mTagBFP2::AID::NLS::tbb-2 3'UTR] II*	this study
jsIs1073 [mec-7p::TagRFP-mito::CBunc119]	Obtained from Michael Nonet
jsIs1073 [mec-7p::TagRFP-mito ,CBunc119]; rbm-26 (gk910) III/hT2 [bli-4(e937) let-?(q782) qIs48] I;III	this study
jsIs609 [mec7p::mtGFP + lin-15(+)] X	Obtained from Michael Nonet
jsIs609 [mec7p::mtGFP + lin-15(+)] X; cueSi36 (Pmec-7::mals-1::scarlet::tbb-2 3'UTR) IV	this study
cueSi35 (Pmec-7::mitoTimer::tbb-2 3'UTR) IV; rbm-26 (gk910) (III)/hT2 [bli-4(e937) let-?(q782) qIs48] (I;III)	this study
cueSi35 (Pmec-7::mitoTimer::tbb-2 3'UTR) IV; rbm-26 (cue23 [rbm-26 (P80L)]) III	this study
*wbmIs67 [eft-3p::3XFLAG::wrmScarlet::unc-54 3'UTR *wbmIs65] V*	CGC
*wbmIs72 [pie-1p::3XFLAG::GFP::unc-54 3'UTR *wbmIs60] III*	CGC
cueSi52(Pmec-7::rbm-26::scarlet::unc-54 3'UTR) IV	this study
cueEx53 (Pmec7::mals-1::tbb2 3'UTR) line #1; jsIs1073 [mec-7p::TagRFP-mito::CBunc119]; muIs32 [mec-7p::GFP + lin-15(+)]	this study
cueEx54 (Pmec7::mals-1::tbb2 3'UTR) line #2; jsIs1073 [mec-7p::TagRFP-mito::CBunc119]; muIs32 [mec-7p::GFP + lin-15(+)]	this study
