# Supplementary material for: Ortholog of autism candidate gene RBM27 regulates mitoribosomal assembly factor MALS-1 to protect against mitochondrial dysfunction and axon degeneration during neurodevelopment
Source: PLoS Biol. 2024 Oct 31;22(10):e3002876. doi: 10.1371/journal.pbio.3002876 (PMC11556708; doi:10.1371/journal.pbio.3002876)
Supplement: S4 Table — (PDF) [file pbio.3002876.s015.pdf]

Primers used in figure 5B
TGCACGAAGGAGCACAAAAC (forward)
CGTGCACCTGAACTCTTTCC (reverse)

Antibodies used in this study

Antibody	Catalog Number	Manufacturer	Dilution
DYKDDDDK Tag Antibody	2368S	Cell Signaling Technology	1:1000
Goat Anti-Rabbit IgG H&L (HRP)	ab205718	Abcam	1: 5000

CRISPR reagents used in this study

CRISPR edit	guide RNA	Homology Directed Repair Template
rbm-26 (P80L)	CACAAGCAAAAGCTATATGC	GCAGTTGCTGAAGCAGAGGTAGGCGCTGTTGATGCAAGCATATAGCTTTTGCTAGTGAGTGCTTCGAAGACTTTGTCGACGAATGGAGCAGT
rbm-26 (L13V)	ATGAGGATGCTCTCTTCGAT	GGCTGTCTAATCAATGAAAAAACTTACATTGGCGAGAGCTCATCAGAGACCCAGTCGAAAAGAGCATCCTCATTGTCGATGTGCATGCTTTCTTTGATATT
rbm-26 (3X FLAG)	ACTAGGACTAATCATTTAGC	ATTTTGAGATCAAAGTTCTAATATAACTAGGACTATTTATCGTCATCGTCTTTGTAGTCGATATCGTGATCTTTGTAGTCGCCATCGTGATCTTTGTAGTCATCATTTAGCAGGTCATCTTCTTCGTCAGACTGAAATCTATCATTTAGCAGGTCATCTTCTTCGTCAGACTGAAATCT
mals-1 (3X FLAG)	AAAGGAAAACATTATCGTCT	CAATAATCTAAATGTTATAAATAACAAAAGGAAAACATTACTTGTCATCGTCGTCTTTGTAGTCGATGTCGTGATCCTTATAATCGCCGTCGTGGTCCTTGTAGTCTCGTCTCGGTGGCAGCAAAATCTTCTGTTTCTCCTCATCAATCTCG
mrpl-58 (3X FLAG)	GCTAATTAGCTAAAACTCAA	GGAAAAATAAAACACGAACAAAATTTCAGCTAATTAGCTACTTGTCATCGTCGTCTTTGTAGTCGATGTCGTGATCCTTATAATCGCCGTCGTGGTCCTTGTAGTCAAACTCAACGGCGGCTCGTCGCGACGCCTTTTTCTCACTCGTTCT
