# Supplementary material for: Ortholog of autism candidate gene RBM27 regulates mitoribosomal assembly factor MALS-1 to protect against mitochondrial dysfunction and axon degeneration during neurodevelopment
Source: PLoS Biol. 2024 Oct 31;22(10):e3002876. doi: 10.1371/journal.pbio.3002876 (PMC11556708; doi:10.1371/journal.pbio.3002876)

Figure 1D Top Panel

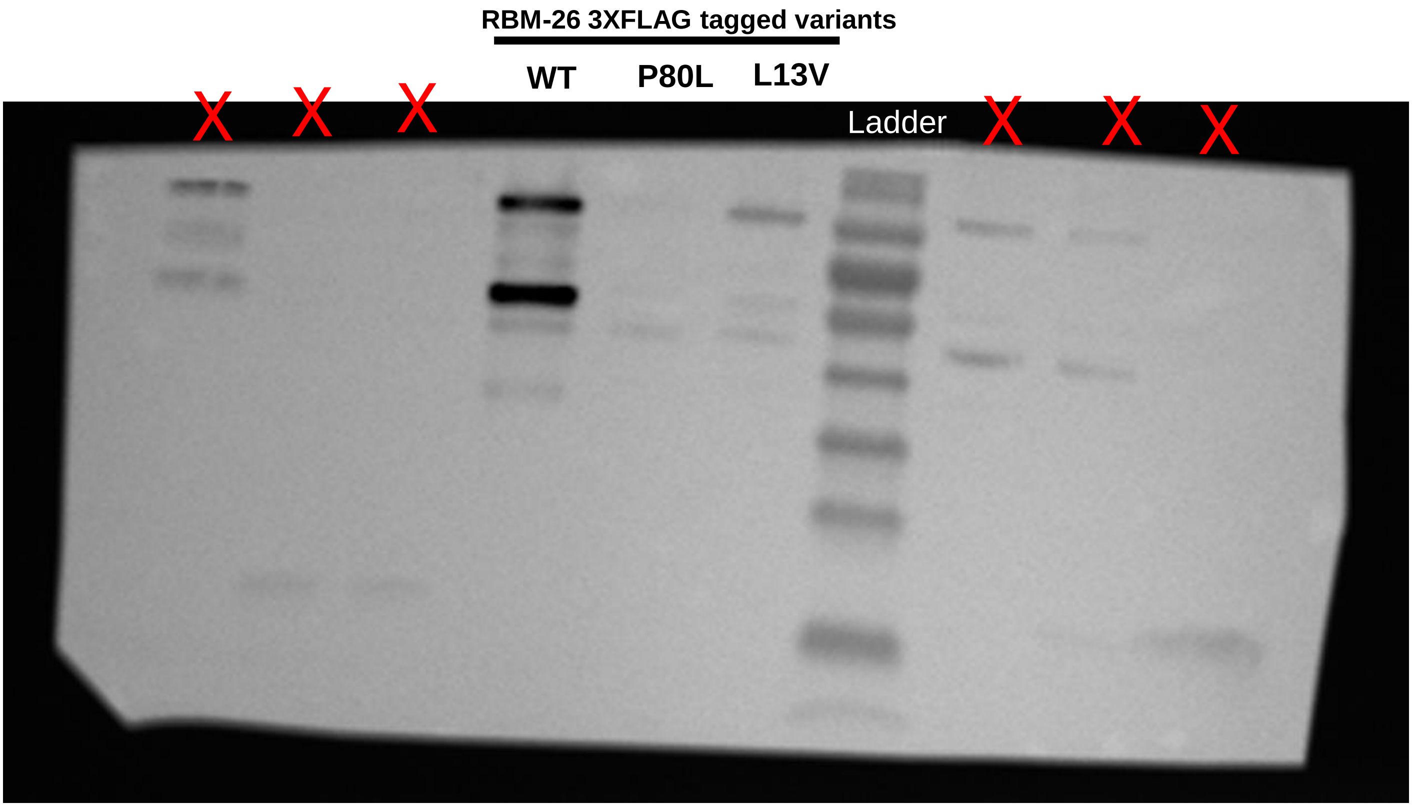

Figure 1D Bottom Panel

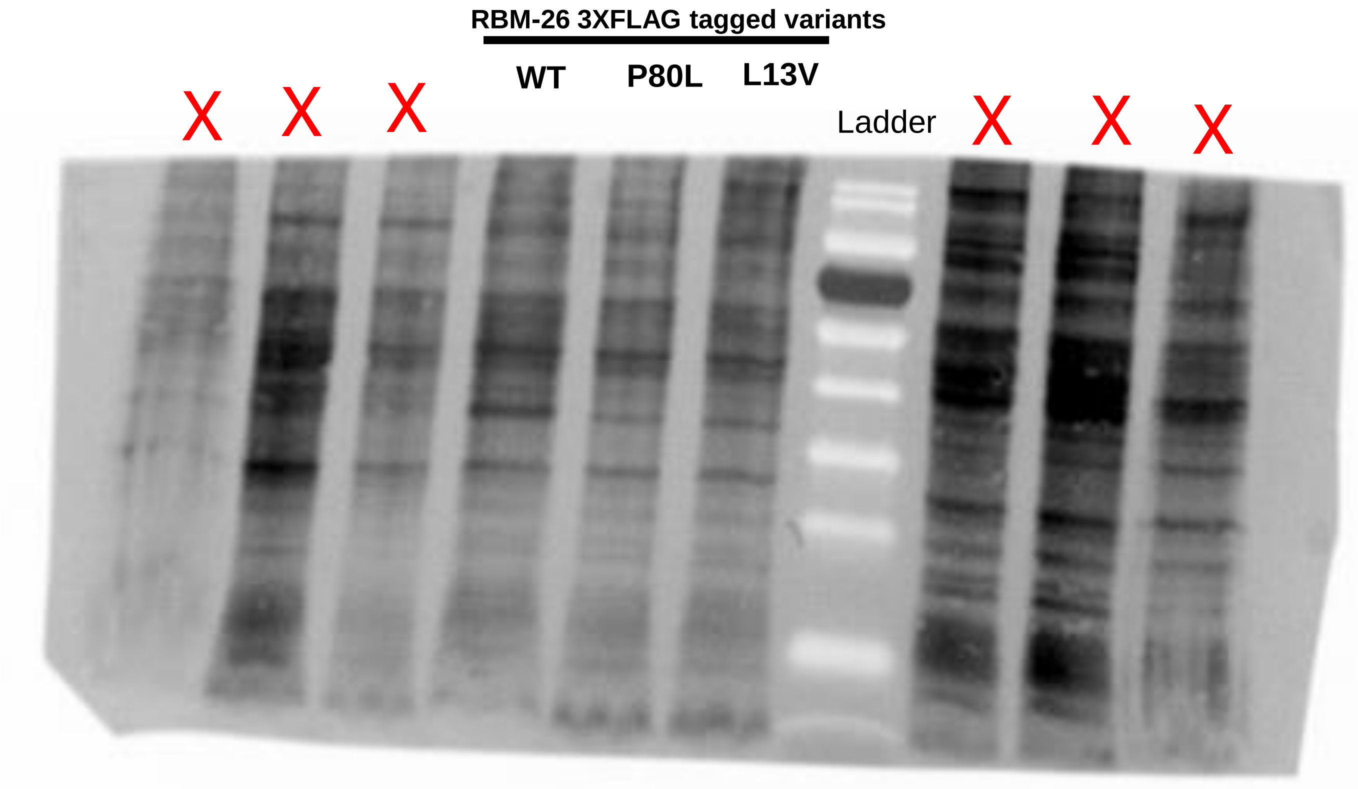

Figure 6C Top Panels

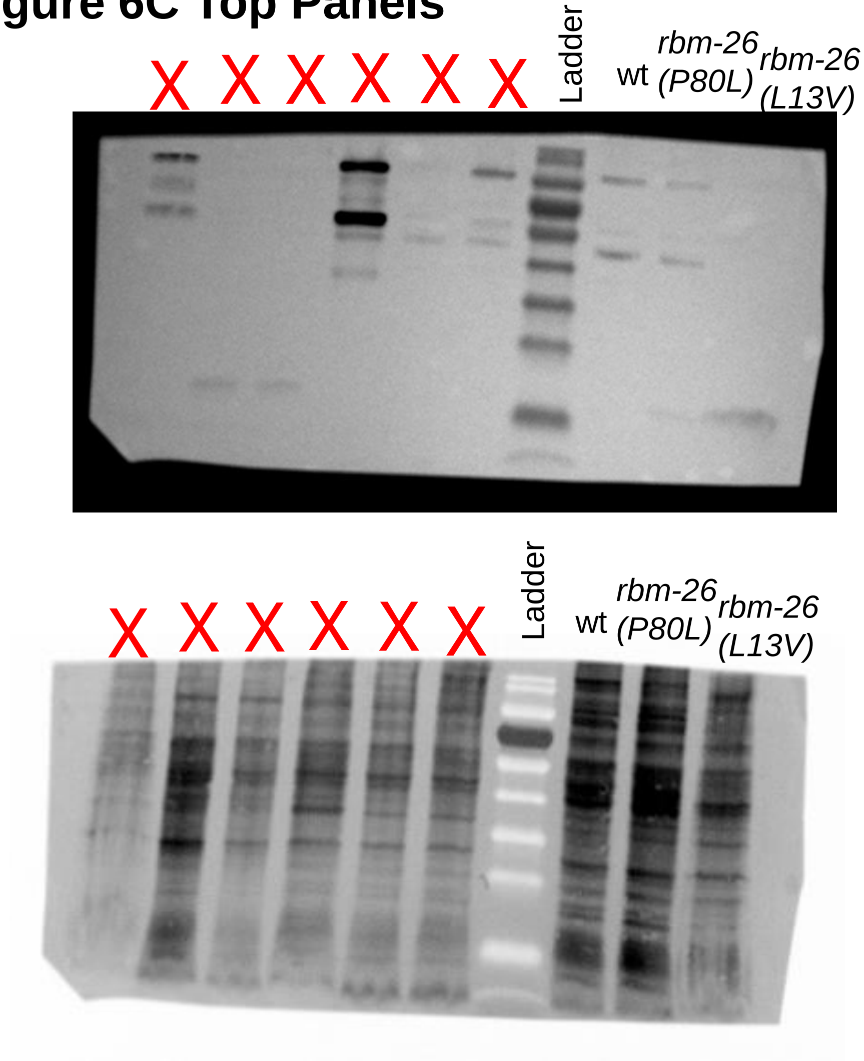

Figure 6C Bottom Panels

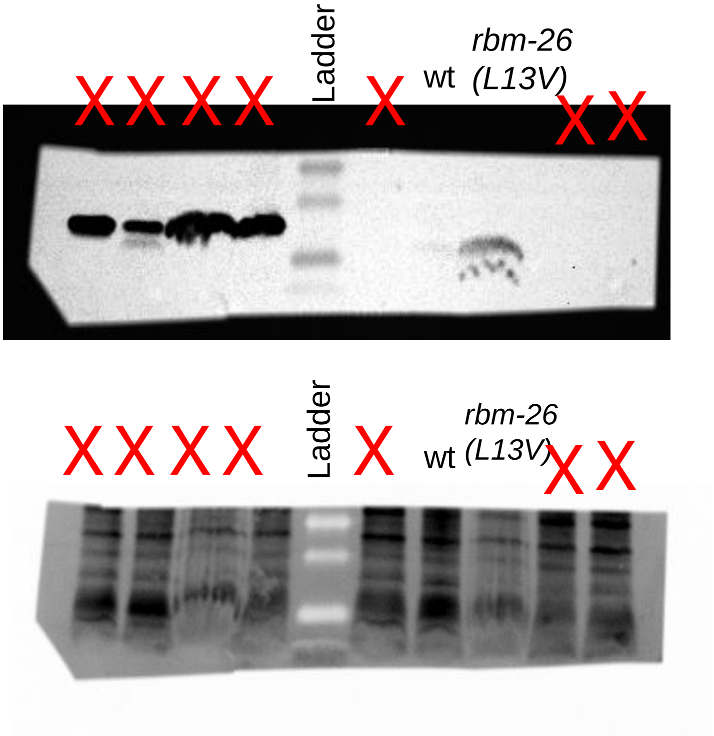

Figure 6C bottom panel

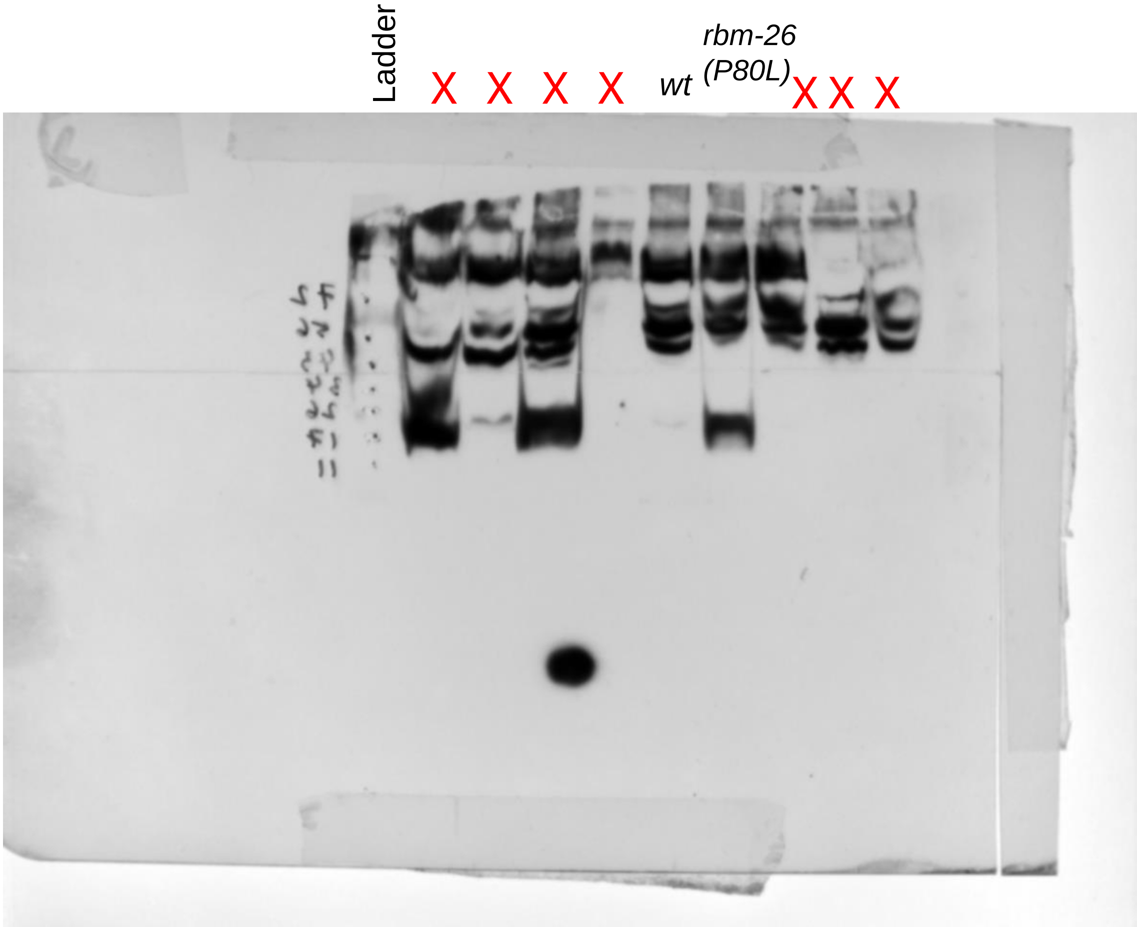

Figure 8D Top Panel

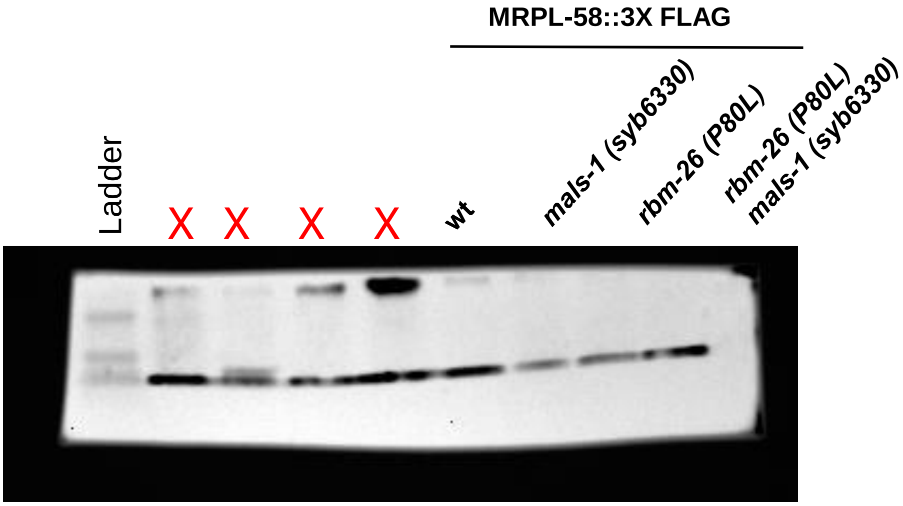

Figure 8D Bottom Panel

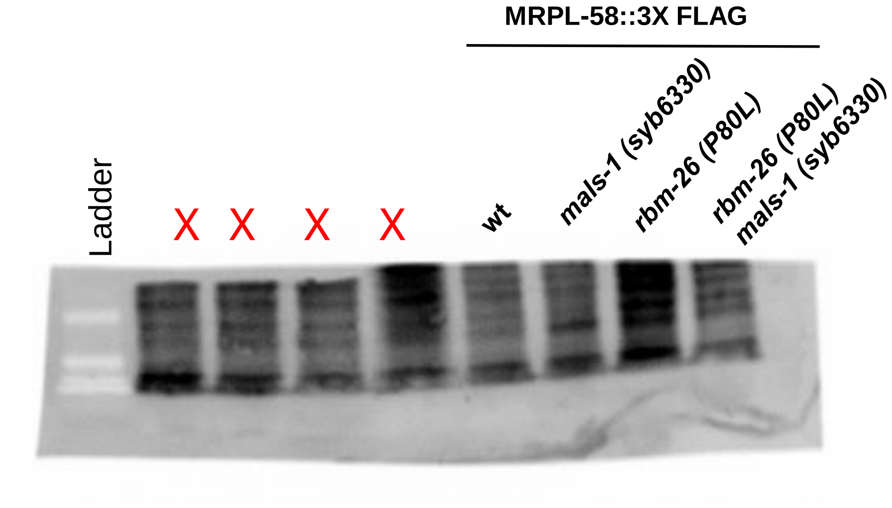

Supplement: S1 Raw Images — (PDF) [file pbio.3002876.s018.pdf]
