# Supplementary material for: Ortholog of autism candidate gene RBM27 regulates mitoribosomal assembly factor MALS-1 to protect against mitochondrial dysfunction and axon degeneration during neurodevelopment
Source: PLoS Biol. 2024 Oct 31;22(10):e3002876. doi: 10.1371/journal.pbio.3002876 (PMC11556708; doi:10.1371/journal.pbio.3002876)
Supplement: Attachment — Submitted filename: 140984_1_rebuttal_3047203_svvctd.pdf [file pbio.3002876.s019.pdf]

Full Revision

Review COMMONS

Manuscript number: RC-2023-02207
Corresponding author(s): Christopher Quinn

[Please use this template only if the submitted manuscript should be considered by the affiliate journal as a full revision in response to the points raised by the reviewers.

If you wish to submit a preliminary revision with a revision plan, please use our "Revision Plan" template. ***It is important to use the appropriate template to clearly inform the editors of your intentions.****]*

1. General Statements [optional]

Please see attached

2. Point-by-point description of the revisions

Please see attached.

General Statement

The reviewers of the prior version of this manuscript found that it had substantial impact and would be of interest to a broad audience. For example, one reviewer said, "This study will be of interest to the autism research community because it bolsters the idea that variants in RBM27 are likely to disrupt gene function and to affect neuronal health. It will also be of interest to the broader cell biology community because it suggests an interesting potential nucleus-to-mitochondria signaling mechanism, in which a nuclear RNA-binding protein might regulate assembly of mitochondrial ribosomes"

Another reviewer said, " I found the manuscript interesting particularly the use of innovative techniques in identifying the target of RBM-26"

However, both reviewers also thought that the work was preliminary. In particular, they said that a more complete analysis of the degeneration phenotypes needed to be done that we also needed to test more alleles, and that we needed to provide rigourous evidence for the cell-autonomous function of RBM-26 in neurons.

We have now completed a thorough analysis of the phenotypes, tested more alleles, and provide multiple lines of evidence for the cell-autonomous function of RBM-26 (transgenic rescue, conditional degradation, and endogenous labeling of RBM-26 expression). We have also addressed all other points of the reviewer with new data. As such, we are confident that our conclusions are now rigorously supported and of high impact.

Reviewer #1 (Evidence, reproducibility and clarity (Required)):

This manuscript uses C. elegans as a model to interrogate the effects of autism-associated variants of previously unknown function in the RNA-binding protein RBM-26/RBM27.

Despite its potential impact, there are several concerns related to the technical rigor and specificity of the observed effects.

Major concerns:
1. The effects on PLM are interesting, but why was this neuron selected for study? Was this a lucky guess or are other axons also affected? It is important to clarify whether the effects of RBM-26 are specific to this neuron or act pleiotropically across many or all neurons. According to CeNGEN, rbm-26 is strongly expressed in the well-characterized neurons ASE, PVD, and HSN. Are there morphological defects in these neurons, or others? As a note, there are also functional assays for these neurons (salt sensing, touch response, and egg laying, respectively).

We have added new data to the supplemental materials showing that loss of *rbm-26* function also causes the beading phenotype in the axons and dendrites of the PVD neuron **(Figure S4 and lines 196-199)**. We have focused on the PLM neuron because our preliminary studies indicated that it had a higher penetrance of axon defects relative to the PVD neuron. Moreover, we observed expression of endogenously tagged RBM-26 in the PLM neuron **(Figure 3A-C and lines 210-215)**.

2. Similarly, the choice of the MALSU homolog seemed like a shot in the dark. It is ranked 46th (out of 63 genes) for fold-enrichment following RBM-26 pull-down, and 9th for p-value. Were any of the mRNAs with greater fold-enrichment or smaller p-values examined further? It is important to determine whether many or all of these interacting genes are overexpressed in the absence of RBM-26 and whether they are also required for the phenotypic effects of RBM-26 mutants, or if the MALSU homolog is special.

We have clarified our reasoning for selecting the MALS-1 ortholog of MALSU1 for further study **(see lines 283-284 and Table S2)**. Amongst binding partners with human orthologs, MALS-1 was by far the top ranked candidate. The adjusted p-value for MALS-1 was 0.0008. The next smallest adjusted p-value was two orders of magnitude larger (0.028 for *dpy-4)*. Moreover, the log2fold fold enrichment for MALS-1 was 1.98, about the same as the largest (ACADS with 2.13). Nonetheless, we agree that some of the other interactors may also be of interest and have thus included them in the supplemental table S2. Although these other potential binding partners are outside the scope of this study, we expect that future studies by ourselves or others may focus on the roles of these other binding partners.

3. In addition to the specificity controls mentioned above, positive and negative controls are needed throughout the results. While each of these may be relatively minor by itself, as a group they raise questions about the technical rigor of the study. Briefly these include:
Fig 1C. Missing loading controls and negative control (rbm-26 null allele). Additional exposures should be included to

show whether RBM-26(P80L) protein or the lower band for RBM-26(L13V) are present at all, relative to the null allele.

We have added no-stain loading controls to figure 1C. We have also switched to using ECL detection, which is much more sensitive and reveals faint bands for RBM-26(P80L) and additional faint bands for RBM-26(L13V). In addition, we have included a longer exposure for the blot **(Figure S1)**. We are unable to test the null, as we can only produce a limited number of small maternally rescued progeny, thereby precluding western blot analysis.

Fig 2. Controls to distinguish overextension of PLM axon from posterior mispositioning of ALM cell body are needed. Quantification of PLM axon lengths in microns (or normalized to body size) with standard deviation, not error of proportion, should be shown. Measurement of "beading phenotype" should be more rigorous, see for example the approach in Rawson et al. Curr. Biol. 2017 https://doi.org/10.1016/j.cub.2014.02.025 . The developmental stage examined, and the reason for choosing that stage, should be described for this and all figures.

We have added new data that shows PLM axon length relative to body length for each of the RBM-26 mutants **(Figure S2 and lines 183-185)**. These results indicate that the PLM axon has a larger axon length to body length ration, suggesting that the PLM/ALM overlap phenotype is a result of PLM axon overextension. For most experiments, we retain penetrance, as this has been standard practice in the field and allows for a much larger sample size (see examples listed below). We have also added examples of how the beading phenotype was measured **(Figure S3)**. Moreover, we have now analyzed this phenotype and others at multiple developmental stages **(Figures 2D-H and Table S1)**. In general, we have conducted experiments at the L3 stage because the *rbm-26(null)* mutants don't survive past this stage. However, for many of our experiments we have also included additional stages as well. We have added this explanation to the methods section of phenotype analysis and also at various locations throughout the text. We have also labeled all graphs to clearly indicate the developmental stages and included.

10.1038/s41467-019-12804-3 Article by laboratory of Brock Grill

10.1371/journal.pgen.1002513 Article by laboratory of Ian Chin-Sang

doi.org/10.1073/pnas.1410263111 Article by laboratory of Chun-Liang Pan

10.1016/j.neuron.2007.07.009 Article by laboratory of Yishi Jin

doi.org/10.1523/JNEUROSCI.5536-07.2008 Article by laboratory of William Wadsworth

Fig 3. Controls without auxin and with neuronal TIR1 expression alone should be included. Controls demonstrating successful RBM-26 depletion, in larvae as well as in embryos at the time of PLM extension, should be included (weak

embryonic depletion might explain why the overextension phenotype is only 14% instead of 40% as in the null). According to CeNGEN, rbm-26 expression in PLM is barely detected, thus depletion with a PLM-specific TIR1 should also be tested. To confirm the authors' identification of the cell marked "N" as the PLM cell body, co-expression of rbm-26 and a PLM-specific marker should be added. Rescue of the rbm-26 mutants with neuronal (and PLM-only) expression should be included to test sufficiency in PLM, and as a further control for potential artifacts of the AID system.

We have added new data showing that an endogenously tagged RBM-26::Scarlet protein is expressed in the PLM neuron **(Figure 3A-C)**. Moreover, we have added rescue experiments, showing that a *Pmec-7::rbm-26::scarlet* transgene can rescue the beading phenotype and the PLM/ALM overlap phenotype **(Figure 3 F-G)**. We have also added controls without auxin **(Figure S7)** and without the *rbm-26::scarlet::aid* gene **(Figure S8)**. We have added a new figure showing auxin-mediated depletion of RBM-26::Scarlet::AID in the PLM neuron **(Figure S10)**. We examined auxin-mediated depletion at the L3 stage for consistency with our auxin-mediated phenotypic experiments. Moreover, these were done at the L3 stage for consistency with other experiments that included the *rbm-26(null)* mutants, which don't survive past this stage.

In general, auxin-mediated knockdown tends to be hypomorphic in neurons. This is likely due to the fact that the neuronal TIR1 driver is expressed at much lower levels relative to the other drivers. In addition, the lower penetrance observed in auxin-mediated PLM/ALM overlap phenotype could reflect the fact that this phenotype resolves by the L4 stage in the hypomorphic mutants. For example, in P80L mutants at the L3 stage we see only about a 20% penetrance of the PLM/ALM overlap phenotype (relative to about 15% in auxin-mediated knockdown).

Fig 4. More rigorous quantification of the distribution of mitochondria along the axon should be included, not only total number, and it should be clarified what region of the axon the images are taken from. Including the AID-depletion strain with and without auxin would further add to the sense of rigor. For the mitoTimer experiments, why is RBM-26(L13V) not included and why do wild-type values differ ~5-fold between experiments (despite error bars being almost non-existent)? A more rigorous approach to standardizing imaging conditions may be needed. Positive controls using compounds that affect oxidation should be included. Measurements of individual mitochondria with standard deviations should be shown, rather than aggregate averages with error of proportion.

We have changed our methodology for measuring mitochondria, so that we now report the density of mitochondria in the axon (number per 100μm), **(Figure 4E-F)**. We agree that this method is much better than counting the total number of mitochondria per axon, as it corrects for differences in body length and axon length). We also now include data for the whole axon **(Figure 4E)**, proximal axon **(Figure 4G)**, and distal axon **(Figure 4H)**. These data suggest that the mitochondrial density defects occur in the proximal axon but not in the distal axon. Using the null allele, we have also examined the timing of mitochondria defects in the axon and report that

the defects begin in the L1 stage and continue throughout larval development **(Figure 4F)**. Individual datapoints have been added for all graphs in Figure 4.

For the mitoTimer experiments **(Figure 5**), we have added data for L13V and have added the individual datapoints to the graph. In the prior version, the values did not differ 5-fold between experiments with the same stage, rather the different graphs were from different stages (as noted in the figure legends/main text) and the L4 stage has much more oxidation than the L2 stage. To clear this up, we have added labels to the graphs to indicate the stages for each experiment. We have also added new data, so that we now show results for the L2, L3, and L4 stages for all three *rbm-26* mutants (see Figure 5C-E). We didn't test the L1 stage because the signal was not sufficient for accurate quantitation.

Fig 5. Additional positive and negative controls should be added, including additional rbm-26 alleles, the AID-tagged strain with and without auxin, and a rescued mutant.

The old Figure 5 has become Figure 6 in the new version. We have added the *rbm-26(L13V)* allele to each experiment, **(Figure 6B-D)**. We have also added the loading controls for the western blot along with quantification for 3 biological replicates of the western blot analysis **(Figure 6D)**. We agree that these additions significantly strengthen the data because they show that two independent alleles of *rbm-26* cause very substantial increase in the expression of *mals-1* at both the mRNA and protein levels. We did not do these experiments with the rescuing transgene or with the AID-tagged strain because these experiments are done on whole worm lysates, whereas the AID-tagged and rescuing transgene are neuron-specific.

Fig 6. Controls showing whether the Scarlet-tagged protein is functional are needed, to rule out dominant negative or toxicity-related effects.

This is Figure 7 in the new version. For this experiment, we are showing that overexpression of MALS-1 does cause defects. The idea is that excessive amounts of MALS-1 causes deleterious effects to the mitochondria. In fact, these defects could be considered as dominant negative or toxic. We considered the possibility of crossing the *Pmec-7::mals-1::scarlet* transgene with *rbm-26; mals-1* double mutants. However, this does not seem workable, because the single copy *Pmec-7::mals-1::scarlet* transgene produces the phenotypes at penetrances that are similar to what we observe in *rbm-26; mals-1* double mutants. We concede that the results of the overexpression experiments in Figure 7 are limited when considered in isolation. However, we think that they are meaningful when considered in combination with the results on the *mals-1;rbm-26* double mutants in Figure 8.

Fig 8. Controls for other mitochondrial components need to be included. It is important to determine if the decrease in ribosomes is specific or reflects a general decrease in mitochondria. If there are fewer mitochondria as suggested in Fig. 4, then of course mitochondrial ribosomal protein levels are also reduced. Additional rbm-26 alleles should be included here as well. Is this effect dependent on the MALSU homolog?

This is Figure 8D-E in the new version. We have added new data showing that the decrease in MRPL-58 expression that is caused by the *rbm-26(P80L)* mutation is dependent on MALS-1. We concede that these experiments cannot be used to determine anything about the mitoribosomes per se, but rather serve as an alternative way of testing the effect of *rbm-26* on mitochondria. We have revised the text accordingly **(lines 355-357)**. Given these limitations we have elected not to try additional mitochondrial markers and have also not included additional *rbm-26* alleles for this experiment.

4. Finally the authors should address concerns about image manipulation, which amplify the concerns about technical rigor outlined above. The image in Fig. 2A appears to have a black box placed over the lower-right portion of the field to hide some features. Black boxes also appear to have been placed over the tops of images in Fig. 4B and 4D and at the left of Fig. 6A, 6B, and 6C. While these manipulations probably do not affect the conclusions, they further undermine confidence in data integrity and experimental rigor.

We have corrected all of these image processing errors. The box in 2A was for the purpose of squaring off a corner that was clipped during image rotation. The boxes in Figures 4 and 6 (of the prior version) were added to give space for labels (without obscuring image features). We have now used alternative methods to accomplish the same goals. For example, in Figures 4-D we have placed the labels outside of the images.

Minor points.

1. C. elegans nomenclature conventions should be followed:

- C. elegans gene names have three or four letters, thus the MALSU homolog cannot be named "malsu-1". Please have new gene names approved by WormBase BEFORE submitting for publication http://tazendra.caltech.edu/~azurebrd/cgi-bin/forms/gene_name.cgi

We have changed *malsu-1* to *mals-1*. In addition, both *mals-1* and *mrpl-58* have now been approved by wormbase and will be listed on the website upon its next update.

- If two sequential CRISPR edits are made on the same gene then they should be listed as a compound allele, such as rbm-26(cue22cue25)

We have updated our gene names to reflect this convention.

- Genes on the same chromosome should not be separated with a semicolon, for example rbm-26(cue40) K12H4.2(syb6330)

We have updated our gene names to reflect this convention.

2. Describing the defects as "neurodevelopmental" is misleading in the case of axon beading or degeneration. Similarly, there is no evidence for an "axon targeting" defect as stated in the abstract.

We have revised such that instead of referring to degeneration phenotypes as neurodevelopmental, we now refer to axon degeneration phenotypes that occur during development. For example, in the abstract we now say, “These observations reveal a mechanism that regulates expression of a mitoribosomal assembly factor to protect against axon degeneration during neurodevelopment.

Regarding targeting defects, this was meant to refer to the misplacement of the PLM axon tip (which contains electrical synapses). However, our subsequent analysis has revealed that these defects are transient in P80L and L13V mutants, as they resolve by the L4 stage. The rbm-26 null axon development defects do not resolve, though these mutant die prior to the L4 stage. Given these findings, we have decided not to use the term of targeting defects. Instead, we now refer to this as an axon tiling defect or PLM/ALM overlap phenotype.

3. In Fig. 5A, the symbol that appears to correspond to F59C6.15 (lowest p-value) is a different size than the others and is colored as ncRNA, whereas WormBase annotates this gene as snoRNA.

This error has been corrected.

4. In the Introduction, the last sentences of the first two paragraphs should be varied ("However, little is known about the [...] mechanisms that protect [...] during neurodevelopment.")

This has been done.

5. Why is RBM-26 protein running as a doublet at both sizes?

We have improved our western blotting methodology by using 12% gel, allowing for better resolution. We have also switched from colorimetric detection to ECL detection, allowing for greater sensitivity. In our new blots, we identify 6 different RBM-26 protein bands. We don’t know the reason for these bands, but speculate that they are the result of post-translational processing **(148-150)**.

6. When showing the RBM-26 expression pattern (Fig. 3) please include a lower-magnification image of the entire animal.

This has been done **(Figure S6)**

7. It is confusing to refer to the RNA IP experiments as an "unbiased screen", which in C. elegans typically refers to a genetic screen.

We now refer to this as a “biochemical screen”.

8. The relationship between axon overextension, beading, and mitochondrial localization is not clear. What causal connection between these is being proposed? The causal connections between these phenotypes, if any, should be clarified experimentally. For example, if the axon extension defects develop before mitochondrial localization defects, then it is unlikely that mitochondrial defects cause axon overextension.

We have added new data showing that the reduction in mitochondrial density within the axon begins during the L1 stage and increases throughout larval development **(Figure 4F)**. We have also added additional data showing that the increase in mitochondrial oxidation is weak in the L2 stage and surges in the L3 stage **(Figure 5C-E)**, coincident with the beginning of the axon degeneration phenotypes. We propose **(lines 383-391)** that a low level of mitochondrial defects is present in L1 larvae, giving rise to the axon tiling defects. In the L3 stage there is a surge in excessive mitochondrial oxidation, giving rise to the axon degeneration phenotypes. We have added a new section to the discussion that addresses the relationship between defects in axon development and axon degeneration **(lines 375-405)**.

9. Please explain how to interpret the difference in axon beading in the two deletion alleles of the MALSU homolog (axon beading defects in tm12122 but not in syb6330). Is syb6330 not a null allele? Or are the defects in tm12122 due to other mutations in this strain background?

One likely reason for this difference is that tm12122 is predicted to cause a partial deletion of the mals-1 coding sequence, whereas the syb6330 is a full deletion. Thus, the tm12122 could be acting as a dominant negative. In fact, prior work on the MALSU1 ortholog has indicated that this protein is subject to interference by a dominant negative construct (see Rorbach et al, Nucleic Acids Res 2012). Nonetheless, we cannot rule out the possibility of a linked second mutation in tm12122. However, since we have found similar phenotypes and genetic interactions with both alleles, we can conclude that these phenotypes and interactions are due to loss of MALS-1, rather than a second mutation.

10. Are mitochondria reduced in number or mislocalized? If they are reduced in number, is this due to altered balance of fission/fusion?

We have adjusted our methods for quantifying mitochondria and have also analyzed the proximal vs distal axon **(Figure 4)**. We find that the density of mitochondria is decreased in the proximal axon, but not in the distal axon. We speculate that this might reflect a higher demand on mitochondria in the proximal axon, due to a higher amount of trafficking activity in the proximal axon **(lines 255-257)**. We propose that the loss of RBM-26 causes dysfunction in mitochondria. Since fission and fusion are mechanisms that can help to repair damaged mitochondria, it is likely that they would be involved in the phenotypes that we observe.

11. In Fig. 3A-D, please keep the labels in the same position in all panels and do not alter brightness settings between single-color and merged panels.

These images have been moved to the supplemental data section **(Figure S5)**. We have adjusted the labels as suggested. We have not changed the brightness settings, as they were already the same in all panels. However, the blue signal in the merged panel does obscure some of the red signal, giving an appearance of an alteration in color balance.

12. The claim that rbm-26 acts cell-autonomously requires PLM-specific depletion and rescue experiments.

We have added new data indicating that a Pmec-7::rbm-26::scarlet transgene can rescue the beading phenotype **(Figure 3F-G)**.

Referees cross-commenting
I appreciate the use of the consultation session to resolve differences between reviewers, but in this case I fully agree with the content and tone of all the comments from the other reviewer -- I think our remarks are very well aligned!

Reviewer #1 (Significance (Required)):

The study engineers autism-associated variants in conserved residues of RBM27 into the C. elegans homolog RBM-26 and identifies neuronal phenotypes potentially relevant to autism and a potential molecular mechanism involving regulation of mitochondrial ribosome assembly.

The key claims of the study are 1} that autism-associated variants in RBM-26 decrease its protein expression; 2} that impaired RBM-26 function leads to a variety of defects in development and maintenance of a single neuron called PLM, including altered axonal localization of mitochondria; 3} that RBM-26 normally binds the mRNA for the C. elegans homolog of MALSU, a mitochondrial ribosomal assembly factor; 4} that loss of RBM-26 leads to overexpression of the MALSU homolog; and 5} that MALSU is required for some of the deleterious effects on the PLM neuron seen in RBM-26 mutants.

This study will be of interest to the autism research community because it bolsters the idea that variants in RBM27 are likely to disrupt gene function and to affect neuronal health. It will also be of interest to the broader cell biology community because it suggests an interesting potential nucleus-to-mitochondria signaling mechanism, in which a nuclear RNA-binding protein might regulate assembly of mitochondrial ribosomes.

My field of expertise is developmental biology in C. elegans.

Reviewer #2 (Evidence, reproducibility and clarity (Required)):

Summary
In this manuscript, the authors studied an ASD-associated gene, rbm-26 in neuronal morphology using the touch receptor neuron PLM in C. elegans, and found that loss-of-function rbp-27 causes overextension and the formation of bulb-like structures in the axon. Using UV-crosslinking RNA immunoprecipitation and RNA-Seq, they identify malsu-1 as a target of rbm-26. Genetic analyses suggest malsu-1 likely functions downstream of rbm-26 in controlling the PLM morphology.
Major comments:

- The authors describe RBM27 is associated with ASD and ID while they only cite SFARI paper that describes a weak association of RBM27 to ASD. The appropriate referenced that show link between RBM27 and ID should be provided.

The link with ID was an error. We had meant to say "ASD or other neurodevelopmental disorders." This has been corrected.

- SFARI database only has three (P79L, R190Q, G348D) mutations listed as ASD-associated. Where are other mutations L13V and R455H, particularly L13V that the authors used to generate the C. elegans mutant come from? Are they associated with intellectual disabilities?

The others came from the devovo-DB. We have added a reference for this database and have also added the primary source references for each of the five de novo variants (see line 121).

- The authors should be very careful when describing 'gene X causes Y diseases'. Many (if not all) of the examples described in this manuscript are disease-associated genes without validation to be causal genes.

We have revised accordingly. For example on lines 433-435, we now say," For example, mutations in the *EXOSC3*, *EXOSC8* and *EXOSC9* are thought to cause syndromes that include defects in brain development such as hypoplasia of the cerebellum and the corpus callosum". We have decided to use the phrase "thought to cause" because three of the five referenced articles on these genes use titles that indicate causation.

- The authors refer PLM axon beading and overextension phenotypes to 'axon degeneration and targeting defects'. The authors must provide additional evidence of axon degeneration (see below). Also the term 'targeting defects' is

misleading as the authors did not examine if overextension of the PLM axon causes targeting defects. At least they should examine some synaptic markers.

To provide more evidence of degeneration we have analyzed several additional phenotypes at multiple developmental stages **(Figure 2 and Table S1)**. Regarding targeting defects, this was meant to refer to the misplacement of the PLM axon tip (which contains electrical synapses). However, our subsequent analysis has revealed that these defects are transient in P80L and L13V mutants, as they resolve by the L4 stage. The rbm-26 null axon development defects do not resolve, though these mutant die prior to the L4 stage. Given these findings, we have decided not to use the term of targeting defects. Instead, we now refer to this as an axon tiling defect or PLM/ALM overlap phenotype.

• Neuronal phenotypes (axon overextension and beading) should be examined at different developmental timepoints (larval, young adult, and aged animals) to test if these phenotypes are indeed degenerative instead of developmental defects.

We have included new data to observe all of these phenotypes at multiple developmental time points **(Figure 2 and Table S1)**.

• The authors use the blebbing (beading) phenotype in the axon as the sole evidence of neurodegenerative properties of the PLM neuron. A more thorough analysis of this phenotype as done by others (Pan PNAS 2006) must be provided to support the authors' claim that this phenotype represents neurodegeneration.

We have included new data on multiple degenerative phenotypes in axons including: blebbing, beading, waviness and breaks **(Table S1).**

• The number of beads per axon should be quantified to better represent the severity of rbm-26 mutant. Individual samples should be plotted in the quantification instead of showing the percentage of animals.

We have added data on the density of beads in rbm-26(null), rbm-26(P80L), and rbm-26(L13V) mutants **(Figure S3).** For most experiments we have decided to use penetrance to measure axon degeneration because this is a standard in the field and allows for a larger sample size. For examples please see:

10.1523/JNEUROSCI.1494-11.2012 (Toth et al, 2012)

https://doi.org/10.1016/j.cub.2014.02.025 (Rawson et al, 2014)

10.1073/pnas.1011711108 (Pan et al, 2012)

https://doi.org/10.7554/eLife.80856 (Czech et al, 2023)

https://doi.org/10.1016/j.celrep.2016.01.050 (Nichols et al, 2016)

• Based on the single gel image in Fig. 1C with no loading control, the P80L mutant appears to have no protein expression. How is the P80L viable while the null mutant is lethal? The authors should quantify the protein expression levels from multiple blots with proper loading controls. If P80L mutation is introduced into RBM-26::mScarlet strain can it cause depletion of the signal in vivo?

We have added new data showing that the RBM-26::Scarlet signal is diminished by the P80L mutation in vivo **(Figure 1E-F)**. We have also added quantification from 3 biological replicate blots **(Figure 1D)**. Finally, we have improved the sensitivity of our blots by using ECL detection and also show various exposures to highlight the fainter bands **(Figures 1C and S1)**. Therefore, we are now able to detect low level expression of RBM-26(P80L) mutant protein. It is likely that the low level of RBM-26(P80L) and RBM-26(L13V) seen on western blots is sufficient to prevent the lethal phenotype.

• 'Moreover, loss of either the SPTBN1 or ADD1 genes causes a neurodevelopmental syndrome that includes autism and ADHD' References are missing, and as described above, be extra careful when indicating causality. Very few genes are known to cause ASD and ADHD.

We have added the citations for this work **(line 81)**. We also note that the titles for both of the cited articles indicate causation. To be on the safe side we have revised this line to say, "Moreover, loss of either the *SPTBN1* or *ADD1* genes are thought to cause a neurodevelopmental syndrome that includes autism and ADHD"

• Fig. 3E F, the authors should use the strains that express TIR1 specifically in the touch receptor neurons to argue cell autonomous function of RBM-26. Alternatively, the authors may conduct PLM neuron-specific rescue experiments to test the sufficiency.

We have added new data indicating that a Pmec-7::rbm-26::scarlet transgene can rescue the beading phenotype and the PLM/ALM overlap phenotype (see Figure 3F-G).

• 'Loss of RBM-26 causes mitochondria dysfunction in axons.' The authors did not examine mitochondria function in axons. They only examined the number of mitochondria, and ROS production in the soma. The authors should provide additional evidence to support the idea that elevated ROS production in the soma is due to mitochondrial dysfunction in axons. Also, the authors should use both P80L and L13V for this experiment, and indicate individual datapoint as dots. Here, they quantified at the L4 stage, which the authors should justify.

We have added the L13V data to this experiment and now show the individual data points. In addition, we have now conducted this analysis at the L2, L3 and L4 stages **(Figure 5C-E)**. We have also revised the text to indicate that loss of *rbm-26* function causes mitochondrial dysfunction in the cell body which could potentially cause a reduction of mitochondria in the axon (see lines 100-101 and 268-270). We

speculate that mitochondria in the axon are also dysfunctional. However, the mitoTimer signal is not bright enough in axons to allow for quantification.

• Figure 5B and C: the authors should also use L13V to quantify malsu-1 mRNA and protein level, and include quantifications in panel C (from multiple blots).

This is Figure 6 in the new version. We have added new data for expression of mals-1 mRNA and protein in *rbm-26(L13V)* mutants **(Figure 6B-D)**. We have also included quantifications from 3 biological replicates **(Figure 6D).**

• In the rbm-26 mutant, the number of mitochondria is reduced, while the amount of MALSU-1 protein is increased. If MALSU-1 is specifically localized at mitochondria in wild type, where does the excessive MALSU-1 go in the rbm-26 mutants? Quantification of MALSU-1 signal intensity should be provided.

Our *Pmec-7::mals-1::scarlet* transgene uses the tbb-2 3'UTR and causes an overexpression phenotype. To address the question posed by the reviewer, we would need to express MALS-1 at endogenous levels. Given that endogenous levels of MALS-1 are very low, it is unlikely that we would be able to visualize its expression. Nonetheless, as a way to address this question we have attempted to create a single copy *Pmec-7::mals-1::scarlet* transgene that utilizes the mals-1 endogenous 3'UTR. We have tried multiple approaches for generating this construct, but all have failed, likely due to sequence complexities within the mals-1 3'UTR. While we cannot say where the extra MALS-1 protein goes, we think that it is likely overloaded into the remaining mitochondria and could also be in the cytosol as well.

• Figure 7C: malsu-1 knockout mutants exhibit PLM overextension phenotype, which is not consistent with their model. The authors should discuss this in detail.

We have added a paragraph to the discussion explaining that mitochondria function could be disrupted by either MALS-1 overexpression or by MALS-1 loss of function **(lines 471-480)**.

• 'To validate these findings, we also repeated these experiments with an independent allele of malsu-1, malsu-1(tm12122) and found similar results (Fig. 7A-C).' The malsu-1(tm12122) exhibits beading phenotype and more severe overextension phenotype which the authors must describe and discuss more carefully.

One likely reason for this difference is that tm12122 is predicted to cause a partial deletion of the mals-1 coding sequence, whereas the syb6330 is a full deletion. Thus, the tm12122 could be acting as a dominant negative. In fact, prior work on the MALSU1 ortholog has indicated that this protein is subject to interference by a dominant negative construct (see Rorbach et al, Nucleic Acids Res 2012).

Nonetheless, we cannot rule out the possibility of a linked second mutation in tm12122. However, since we have found similar phenotypes and genetic interactions with both alleles, we can conclude that these phenotypes and interactions are due to loss of MALS-1, rather than a second mutation (albeit at a slightly different penetrance). We have added these considerations to the results section **(lines 342-244).**

• Figure 8: The authors should include data from L13V, malsu-1 and rbm-26; malsu-1 mutants. Quantification from multiple blots should be provided.

This is Figure 8D in the new version. We have added the malsu-1 and rbm-26;malsu-1 double mutants to this experiment. We have also added quantification from multiple biological replicate blots. As pointed out by the other reviewer, we think that this experiment does not give specific information about mitoribosomes, but is an alternative approach to looking at the reduction in mitochondria. Given this limitation and considering that we have added L13V data to the mitochondria experiment in Figure 8B, we have elected not to add additional data on L13V to the western blot experiment in Figure 8D

Minor comments:

• 'Consistent with a role for mitochondria in neurodevelopmental disorders, some of these disorders include a neurodegenerative phenotype.' Why is it consistent to have neurodegenerative phenotypes if mitochondria is associated with neurodevelopmental disorders? A better explanation would help.

We have changed this sentence to, “Some neurodevelopmental syndromes feature neurodegenerative phenotypes that occur during neuronal development.”

• L13V is generally more severe in axon overextension phenotype than P80L while protein level is more abundant. The authors should discuss about this.

We have also added a time course for the PLM/ALM overlap phenotype mutants **(Figure 2D)**. This new data shows that the PLM/ALM overlap is quite similar overall between the P80L and L13V mutants. Both of these mutations cause an increase in PLM/ALM overlap in early larval development that is resolved by the L4 stage. The P80L phenotype resolves slightly sooner for reasons that are unknown. This could reflect differences in expression within the PLM that are not reflected in the whole worm lysate. This could also be due to a slight difference in the genetic background or other stochastic factors. The key point is that these two independent alleles cause similar phenotype overall, indicating that this phenotype is the result of loss in RBM-26 function.

• Fig. 2E, F: 'Beading refers to focal enlargement or bubble-like lesions which were at least twice the diameter of the axon in size.' How are the diameters of axons measured? A more detailed quantification method, and examples of measurement should be provided.

We have added example measurements to the supplemental section **(Figure S3)**. Additional detail on the measurements are in the Methods section **(lines 517-518)**.

• Figure 3: The authors should also include low-magnification images to show where RBM-26 is expressed. The current image does now allow identifying cells. The transgene that labels the nuclei of hypodermis should be indicated in the manuscript. Specifically, the expression of the RBM-26 in the PLM should be shown.

We have added a low magnification image **(Figure S6)** and have also added images of endogenously tagged RBM-26:Scarlet in the PLM (**Figure 3A-C)**. The transgenic label for the hypodermis has been added to the legend of Figure S5.

• Figure 3: 'Tissue specific degradation of RBM-26::SCARLET::AID was achieved due to cell-type specific TIR-1 driver lines (see methods for details).' This information is not provided in the method section.

This information has been added to methods section, ”Auxin proteindegredation”

• Fig. 4 E. Values from individual samples should be indicated as dots. Representative images of P80L and L13V should be included. Conduct quantifications at adult stage as the authors use in other quantifications, or justify use of specific developmental stage (L3) they used.

Figure 4 has become Figures 4 and 5 in the revised version. We have updated the graphs to include dots for individual data points. We have added quantifications of the mitoTImer experiments for the L2, L3 and L4 stages **(Figure 5C-E)**. We note that our other experiments were done at the L1, L2, L3 and L4 and adult stages. The mitoTimer signal is not sufficient at the L1 stage for quantification. At the adult stage, the red signal becomes saturated. We have added representative images for mitoTimer in P80L and L13V mutants **(Figure S9)**.

• The genes malsu-1 and mrpl-58 are not listed on wormbase. If the authors would like to designate names to these gene, they should clearly indicate that along with the sequence name.

We have changed *malsu-1* to *mals-1*. In addition, both *mals-1* and *mrpl-58* have now been approved by wormbase and will be listed on the website upon its next update.

• The authors found that MRPL-58 amount is reduced in rbm-26 mutants (which require additional verifications). This can be explained by the fact that axonal mitochondria number is reduced in the rbm-26 mutants. How did the authors confirm that the reduction in MRPL-58 level is due to the disruption of mitoribosome assembly?

This is Figure 8D-E in the new version. We have added new data showing that the decrease in MRPL-58 expression that is caused by the *rbm-26(P80L)* mutation is

dependent on MALS-1. We concede that these experiments cannot be used to determine anything about the mitoribosomes per se, but rather serve as an alternative way of testing the effect of *rbm-26* on mitochondria. We have revised the text accordingly **(lines 355-357)**.

• 'MALSU-1 is a mitoribosomal assembly factor that functions as part of the MALSU1:LOR8F8:mtACP anti-association module [37-39].' I don't think these are known for C. elegans MALSU-1.

We have revised to, “MALS-1 is an ortholog of the MALSU1 mitoribosomal assembly factor that functions as part of the MALSU1:LOR8F8:mtACP anti-association module”

• 'Moreover, our results also suggest that disruption of this process can give rise to neurodevelopmental disorders.' I feel this is a quite a bit of stretch.

This has been replaced with, “Therefore, we speculate that human RBM26/27 could function with the RNA exosome complex to protect against neurodevelopmental defects and axon degeneration in infants.” **(lines 371-373)**

Referees cross-commenting
Yes, many of our comments overlap, and I fully agree with all comments from the other reviewer too.
Reviewer #2 (Significance (Required)):

I found the manuscript interesting particularly the use of innovative techniques in identifying the target of RBM-26, The genetic analyses of rbm-26 and malsu-1 generally support the authors main conclusions that rbm-26 inhibits malsu-1 and be of potential interest to basic neuroscientists and cell biologists. However, the current manuscript looked premature which made my reading experience less pleasant. The phenotypic analyses is superficial compared to works similar to this work, which are insufficient to support the authors' claim of 'axon degeneration and targeting defects'. A number of issues listed above should be addressed before this manuscript is published.
The reviewer's expertise: neurodevelopment in model organisms.
