# Supplementary material for: Ortholog of autism candidate gene RBM27 regulates mitoribosomal assembly factor MALS-1 to protect against mitochondrial dysfunction and axon degeneration during neurodevelopment
Source: PLoS Biol. 2024 Oct 31;22(10):e3002876. doi: 10.1371/journal.pbio.3002876 (PMC11556708; doi:10.1371/journal.pbio.3002876)
Supplement: Attachment — Submitted filename: Reviewer Response.docx [file pbio.3002876.s021.docx]

REVIEWS:

Reviewer #1: The authors have added some additional controls that improved the manuscript.  There are still several issues related to experimental rigor and data integrity.

1. Images altered with black boxes
As noted in the previous review, the image in Fig. 2A has been altered by using a black box to cover the bottom right corner of the image.  In their rebuttal letter the authors said they corrected this, but the black box is still there.

The black box in Fig 2A was added to square off a corner that had been clipped during rotation. We have corrected this issue by cropping the image to remove the clipped corner.

2. Protein size markers altered
In the previous submission the major RBM-26 bands were shown to be migrating at ~72 kD and ~50 kD.  They are now shown as migrating at >95 kD and ~60 kD.

We had improved our size estimates by allowing the gel to run for a longer period of time. Thus, we think that the sizes are now more accurate relative to the first version. As required by journal policy, we are also including the uncropped image with protein size markers in the data archive.

3. mals-1::Scarlet
The authors do not address the concern from the previous review that the phenotypes caused by mals-1::Scarlet in Fig. 7 (previously Fig. 6) are an artifact of the Scarlet tag.  The concern is that the Scarlet tag creates a dominant or toxic product and the phenotypes would not be seen with expression of untagged mals-1.  The authors should clarify in the text that the observed defects may be artifacts of the Scarlet tag and not representative of normal mals-1 activity.

We have redone the experiment shown in Figure 7D-F. In the new version, we have used mito::RFP to observe mitochondria and two independent extrachromasomal arrays to express Pmec-7::mals-1 (without Scarlet tag).

4. Discrepancy with mals-1(tm12122)
The authors also do not address the previous concern that the phenotypes for mals-1(tm12122) are stronger than the putative null mals-1(syb6330).  In their rebuttal letter they say that "tm12122 could be acting as a dominant negative" to impair function of the wild-type copy, but this strain is homozygous and in any case this would not explain why tm12122 is worse than null.  In the text they "speculate that this might be the result of an incomplete deletion of the sequence coding for MALS-1 in the syb12122 [sic] allele" but they need to add that it also may be due to other mutations present in the strain that are not related to mals-1.

We have added, "...which might create a truncated protein product that could interfere with the functions of proteins that normally interact with MALS-1. Alternatively, this difference could be caused by a difference in the genetic background." (see lines 362-365).

5. Axon overlap defects
The authors now show that the axon overlap, which was a focus of the previous version, is actually transient.  I am not sure what it means when they say these defects "resolve" by L4 - it is unclear to me if this is by shortening of the axon or some other mechanism. It remains confusing why loss of mals-1 and overexpression of mals-1 both cause this phenotype, while loss of mals-1 suppresses the other rbm-26 phenotypes.  In general I am not sure what to make of this transient defect that seems to be caused by many different manipulations of rbm-26 and mals-1.  

We have added additional information on the axon overlap phenotype from Galegos, 2004 (see lines 160-167). This reference shows that in wildtype, the PLM and ALM axons overlap during the L1 stage. This overlap is resolved through changes in the PLM growth rate. We suggest that *rbm-26* mutations cause a delay in the resolution of the overlap phenotype (see lines 188-190).

Regarding the overexpression of MALS-1 and loss of MALS-1 phenotypes, we have added, " Although our work focuses on mitochondrial and axon defects caused by excessive expression of MALS-1, we also observe decreased mitochondrial density and axon overlap defects in *mals-1* loss of function mutants. These observations are consistent with the role of MALSU1 as a mitoribosomal assembly factor in mammalian cells {Rorbach, 2012 #235}. Thus, loss of MALSU-1 or MALS-1 expression would be expected to cause defects in mitoribosomal assembly. We have also found that overexpression of RBM-26 can cause axon overlap defects. Although we do not know how overexpression of RBM-26 causes this phenotype, this observation could reflect repression of MALS-1 expression by overexpression of RBM-26. Overall, these observations suggest that mitoribosomal assembly could be disrupted by either loss of MALS-1 or excessive expression of MALS-1." (see lines 480-490)

6. Incomplete TIR1 effects
The authors state, "We also note that both of these phenotypes occurred at a lower penetrance in these degradation experiments relative to experiments with the rbm-26 mutant alleles, suggesting incomplete degradation of the RBM-26::Scarlet::AID protein."  They need to mention that it may also be because rbm-26 acts from other cell types.

We have added, "However, given the widespread expression of RBM-26, we cannot exclude the possibility that it might also have non-autonomous functions (see lines 244-245).

7. Speculation
In my view the speculation goes so far beyond the data that it is inappropriate.  Examples include:
- " the degenerative and developmental defects caused by loss of rbm-26 function in C. elegans larvae are reminiscent of defects in human infants who carry mutations in various components of the RNA exosome"

-The paragraph containing this statement has been deleted.

- "we speculate that RBM27 may function with the RNA exosome in humans to protect against neurodevelopmental disorders."

-This has been deleted.

- "We speculate that mitochondria in the proximal PLM axon might be more susceptible to damage because of the higher level of axon transport that occurs in the proximal PLM axon"

-This has been deleted.

- "In later larval stages, the mitochondrial damage becomes substantially worse, giving rise to axon degeneration." (Why not that axon degeneration damages mitochondria?)

We have found that axon degeneration in *rbm-26* mutants can be suppressed by loss of a mitoribosomal assembly factor that is excessively expressed in *rbm-26* mutants. Therefore, we think that the most likely scenario is that mitochondrial damage causes axon degeneration in rbm-26 mutants.

- The paragraphs on MTREC/PAXT and cerebellar hypoplasia etc. The connection is interesting but there is no experimental footing in this study.

We have revised this section to clarify that our study does not address the mechanism of how RBM-26 regulates MALS-1 and that we are providing information on the ortholog's interaction with PAXT and MTREC as a way to think about a possible mechanism for RBM-26. We think that this information is important because it helps to provide a possible mechanism through which RBM-26 could negatively regulate expression of MALS-1. We agree that this section was too long and have condensed it down to a single paragraph.(see lines 421-434).

- Similarly the section on mitoribosomes is way beyond what is supported by the data, especially given the authors' rationale for not including key controls ("these experiments cannot be used to determine anything about the mitoribosomes per se, "Given these limitations we have elected not to try additional mitochondrial markers and have also not included additional rbm-26 alleles for this experiment").

We have revised this section to make it clear that our study does not identify the mechanism through which excessive MALS-1 disrupts mitochondrial function and that we are presenting information on mitoribosomes as a way to consider a potential mechanism. In fact, this information is critical when thinking about why both MALS-1 overexpression and loss of MALS-1 function would cause mitochondrial defects. (see lines 459-464).

Reviewer #2: In the revised manuscript, the authors conducted detailed phenotypic analyses at different larval stages and found that the PLM axon overextension phenotype, which the authors now describe as ALM/PLM overlap, is only transiently observed during L2-L3 stages in the disease-associated rbm-26 mutants. The authors also examined the degeneration phenotypes in the PVD sensory neuron, where disease-associated rbm-26 mutants exhibited statistically insignificant defects in most aspects. 
Overall the authors added substantial new datasets, and significantly improved the rigidity of their work, while new dataset also revealed the effect of rbm-26 mutations to be more transient and subtle than originally described. 
In addition, a number of issues, some of which have been raised in the original reviews, still remain in the current version of the manuscript. 
1. Definition of neurodegenerative phenotypes during neurodevelopment. 
The authors addressed the concerns of both reviewers about mixing developmental and degenerative phenotypes by defining the degenerative phenotype during larval development. However, in the revised manuscript, the authors mix up the terms 'larval development' and 'neurodevelopment' throughout, including their title, which still remains a major cause of confusion. Regarding the PLM/ALM overlap phenotype during L2-L3 stages, the authors called 'developmental defects', which contradicts previous work demonstrating that the PLM neuron completes its axon development in the first larval stage (Gallegos and Bargmann 2004).

The work cited above by Galegos and Bargmann indicates that growth of the PLM does not stop during the first larval stage, but rather continues throughout the entire period of larval development. Therefore, rbm-26 mutants cause PLM axon degeneration that occurs while the PLM axon is still developing. To help clarify this issue, we have added "The PLM axon grows in three phases {Gallegos, 2004 #347}. The first phase occurs during the first part of the L1 stage when the axon grows rapidly and overshoots the ALM neuron, thereby creating a transient PLM/ALM overlap. The second phase occurs during the later L1 stage, when PLM axon growth pauses, allowing for the resolution of the PLM/ALM overlap. The third phase of PLM axon growth occurs throughout the remainder of larval development and features continuing axon growth that matches the speed of body growth." (see lines 160-167).

2. handling of data that is inconsistent with the authors' model. 
In the original review, I questioned some data that were inconsistent with the authors' model. Unfortunately, the authors only responded to these in their rebuttal without providing additional statements in the result section in their manuscript. The authors should not make arguments just by using datasets that are consistent with their models. Specific datasets are listed below: 

Figure 3F. I did not find authors explaining why rbm-26 overexpression causes axon overlapping phenotype.

We have added the following to the results section," We note that overexpression of RBM-26::Scarlet also caused a low penetrance of PLM/ALM axon overlap defects, suggesting that excessive function of RBM-26 may also cause some defects in axon development" (lines 244-245). In the discussion we have added, " We have also found that overexpression of RBM-26 can cause axon overlap defects. Although we do not know how overexpression of RBM-26 causes this phenotype, this observation could reflect repression of MALS-1 expression by overexpression of RBM-26."(see lines 485-488)

Figure 8A. The authors did not observe suppression of rbm-26 (L13V) by mals-1 mutation, but this was not described nor discussed.

We have added the following to the results, "We also repeated these experiments with the *rbm-26(L13V)* mutation and found similar results, with the exception that suppression of the beading phenotype by loss of *mals-1* did not quite reach statistical significance (p=0.1)." (lines 352-354).

line 335-6: 'because mals-1(syb6330) single mutants already have this phenotype at a penetrance equal to rbm-26(P80L) mutants.' This should be more carefully explained in the result section as this contradicts the observation of the mals-1 overexpression phenotype in Figure 7.

We have revised this statement as follows, "because *mals-1(syb6330)* single mutants already have this phenotype at a penetrance equal to *rbm-26(P80L)* mutants, suggesting that loss of *mals-1* function can also cause the axon overlap phenotype."(lines 346-348) We have also added the following to the same paragraph," In addition, we noted that the *rbm-26(L13V)* mutation did cause axon overlap defects that were greater than what was observed in the *mals-1(syb6330)* and we found that these defects were suppressed in *rbm-26(L13V) mals-1(syb6330)* double mutants **(Figure 8C)**. In addition, we have added the following to the discussion," Although our work focuses on mitochondrial and axon defects caused by excessive expression of MALS-1, we also observe decreased mitochondrial density and axon overlap defects in *mals-1* loss of function mutants."

Figure 7F. wildtype mitochondria number in this panel is around 6, which is similar to the rbm-26 mutants in Figure 4E. The discrepancy is not explained. 

This discrepancy was caused by using different mitochondrial markers and has been corrected in the current version. In the prior version, for Figure 4E, we used the jsIs609 transgene (expresses mitoGFP) for the experiments in figure 7F. For Figure 4E (and all other mitochondrial density experiments), we used the jsIs1073 transgene (to express mitoTagRFP). We have found that the jsIs609 transgene has a negative effect on mitochondrial number. To solve this problem, we have redone the experiments in Figure 7F using the jsIs1073 transgene (and also with an untagged version of MALS-1).

3. The definition of 'causality'
In the revised manuscript, I still see the authors using the term 'causative' throughout. The authors responded to my previous comment by stating, 'We also note that the titles for both of the cited articles indicate causation.'  The authors should carefully read each cited article to understand what previous work did rather than relying on the title of the papers. Examples are listed below:

We think that the evidence presented in these references is most consistent with causality. This is because the authors conducted pedigree analysis of patients exhibiting specific syndromes. Moreover, similar phenotypes were observed in animal models. However, we do recognize the caveats to this conclusion and have revised as follows:

"*EXOSC3* or *EXOSC9* gene function has been associated with a neurodevelopmental syndrome"

"loss of either the *SPTBN1* or *ADD1* genes have been associated with a neurodevelopmental syndrome "

"Mitochondrial dysfunction has been associated with neuronal degeneration."

Line 66, 71: 'Consistent with a causative role for mitochondria dysfunction in neurodevelopmental disorders' As pointed out in the original review, the authors should pay extra attention when referring to the causal effects. All examples the authors listed in this paragraph only suggest correlation not causation.

Most of this paragraph refers to correlations. For this reason, the paragraph begins with,"Emerging evidence suggests an association between mitochondria and neurodevelopmental disorders". However, the sentence in question was referring to a specific experiment that suggests causation in mice. To further emphasize that the evidence for causation is in mice we have modified the sentence as follows: "In mice, a hypomorphic missense mutation in the *ND6* mtDNA gene exhibit autism endophenotypes and also produce excess ROS in the brain " (lines 65-67).

line 399-400: 'despite causing severe intellectual disability in humans [41]' The referenced work the authors cited only confirmed the effect of these mutations in C. elegans, similar to this manuscript. Seeing mutant phenotype in C. elegans does not prove the causal effect of these mutations in humans. 

We have added the reference for the related human work and also changed the word caused to implicated (see line 412-413).

Other comments are listed below:
Line 51 (and many other places): 'Axon tiling - PLM/ALM overlap phenotype' It is confusing to use two terms interchangeably when describing one phenotype. I suggest that the authors use the PLM/ALM overlap phenotype. 

We have changed these to axon overlap defects.

line 188-189: Table S1. The degeneration phenotypes, except for the axon beading phenotype, are very minor, with no statistical analyses provided.

We have added statistical analysis to Table S1. We have also noted the subtle nature of these phenotypes in the text, "In addition to beading we observed several other more subtle degeneration phenotypes **(S1 Table)**, including axon breaks **(Figure 2G,H)**." (Lines 201-202)

line 198: 'we also observed the PVD neuron and observed signs of degeneration in both its axon and dendrites' The authors should note that the phenotype is very subtle and most of them are statistically insignificant.

In the prior version, we neglected to denote statistical analysis on most of the PVD phenotypes. We have now added those to Panel C of S4 Fig (3 of 5 phenotypes are subtle, yet statistically significant). We have also noted in the text that these are subtle.

line 295: Figure 6C. Please include the original gel image, as the presented panel does not even include entire MALS-1 protein bands.

For Figure 6C, we have redone the image cropping to show the whole MALS-1 band. We have also added the whole uncropped gel image to the archive as per journal policy.

line 334: Figure 8B. The color-coding of this panel is confusing. Please use consistent color coding across figures in this paper. The datasets for wild-type, P80L and L13V appear to be identical to the ones in Figure 4E. If the same data were reused, those should be stated in the figure legend.

We have redone the color coding for Figure 8B and have also added note to the figure legend indicating the reuse of data sets from Figure 4E.

line 343-4: 'speculate that this might be the result of an incomplete deletion of the sequence coding for MALS-1 in the syb12122 allele.' this is not understandable without reading what the authors described in their rebuttal. The authors should describe in the result or discussion section why they think incomplete deletion in the tm12122 allele causes a more severe phenotype compared to a null mutant. The authors' argument of tm12122 being dominant negative is also misleading as dominant negative cannot be more severe than a null mutant unless there are paralogs of MALS-1.

We have added the following, "We noticed that this *mals-1(tm12122)* allele had slightly higher penetrance of defects relative to the *mals-1(syb6330)* mutant and speculate that this might be the result of an incomplete deletion of the sequence coding for MALS-1 in the *tm12122* allele, which might create a truncated protein product that could interfere with the functions of proteins that normally interact with MALS-1. Alternatively, this difference could be caused by a difference in the genetic background." (lines 360-365)

Line 113-114 'we speculate that RBM27 may function with the RNA exosome in humans to protect against neurodevelopmental disorders.' I would remove this from the introduction as it is purely speculative and has no experiment associated with this.

This has been removed.

There are also a number of typos that the authors should carefully check.

This has been done.
